# A Shortest-Path-Based Method for the Analysis and Prediction of Fruit-Related Genes in *Arabidopsis thaliana*

**DOI:** 10.1371/journal.pone.0159519

**Published:** 2016-07-19

**Authors:** Liucun Zhu, Yu-Hang Zhang, Fangchu Su, Lei Chen, Tao Huang, Yu-Dong Cai

**Affiliations:** 1 School of Life Sciences, Shanghai University, Shanghai, People’s Republic of China; 2 Institute of Health Sciences, Shanghai Institutes for Biological Sciences, Chinese Academy of Sciences, Shanghai, People’s Republic of China; 3 College of Information Engineering, Shanghai Maritime University, Shanghai, People’s Republic of China; Tianjin University, CHINA

## Abstract

Biologically, fruits are defined as seed-bearing reproductive structures in angiosperms that develop from the ovary. The fertilization, development and maturation of fruits are crucial for plant reproduction and are precisely regulated by intrinsic genetic regulatory factors. In this study, we used *Arabidopsis thaliana* as a model organism and attempted to identify novel genes related to fruit-associated biological processes. Specifically, using validated genes, we applied a shortest-path-based method to identify several novel genes in a large network constructed using the protein-protein interactions observed in *Arabidopsis thaliana*. The described analyses indicate that several of the discovered genes are associated with fruit fertilization, development and maturation in *Arabidopsis thaliana*.

## Introduction

In botany, fruit usually refers to the seed-bearing structure in angiosperms and results from the maturation of flowers, the gynoecium of which contributes to the formation of all fruit structures. The whole development and maturation processes of fruits are precisely regulated by functional genes and can be artificially divided into three main steps: fertilization, development and maturation. During the fertilization process, activation of the gibberellin signaling pathways contributes to fruit growth [1], and genes such as DELLA, ARFs and Aux/IAA have been reported to participate in the gibberellin signaling pathways and to contribute to both maintenance of the hormonal balance and the regulation of cell division and size alteration, functions that are crucial in the fertilization process [2, 3]. In addition to the fertilization process, the development stage of fruit also involves various functional genes. Fruits can be divided into fleshy and dry fruits, and the development of these different types of fruits involves different pathways and genes. For example, the development of tomato, as a fleshy fruit, involves cell division and expansion of the ovary tissues, and several genes contribute to these processes, including Fw2.2, which is responsible for approximately 30% of the variation in tomato size and contributes to the regulation of the size of fleshy fruits [4]. In addition, genes such as SUN, OVATE and FASCIATED in tomato are associated with the shape of the tomato fruit during its development [5–7]. Thus, the fertilization and development processes of fruits (including tomato) are precisely regulated. The maturation process of dry fruits can be further separated into two stages, namely maturation and dehiscence, whereas that of fleshy fruits can be separated into maturation and ripening. During the maturation and dehiscence of dry fruits, genes such as SHP1 and SHP2 are positively regulated by the IND gene, which further contributes to the maturation process. All three genes (SHP1, SHP2 and IND) are further regulated by a core gene for fruit maturation, FRUITFULL (FUL). The fertilization, development and maturation processes of fruits are all regulated by specific genes, implying the need to identify functional regulatory genes that contribute to the fruit-associated pathways activated during the fertilization, development and maturation processes of fruits.

*Arabidopsis thaliana* (also known as thale cress, mouse-ear cress or Arabidopsis) is a small cruciferous plant that originated from Eurasia [8]. *Arabidopsis thaliana*, which was first named *Pilosella siliquosa*, was first discovered in 1577 in the Harz Mountains in Germany by *Johannes Thal*, a German botanist [9]. During the 300 years following its discovery, *Arabidopsis thaliana* was identified and confirmed to be native to Europe, Asia, and northwestern Africa [8]. In 1943, *Friedrich Laibach* first proposed *Arabidopsis thaliana* as a model organism. Since then, the application of *Arabidopsis thaliana* in biological sciences, particularly the fields of mutation and genomics, has gradually increased and become popularized [10, 11]. *Arabidopsis thaliana* presents four main advantages regarding its use as a classical and functional model in the biological sciences, particularly genetics. First, *Arabidopsis thaliana* is short (7–40 cm), which enables scientists to grow the plant on a large scale in a small amount of space. Second, *Arabidopsis thaliana* has a strong reproductive ability [12], which permits the generation of a large number of plant seedlings within a short time and reduces the cost of experiments. Additionally, as *Arabidopsis thaliana* is a self-pollinated plant, its fertilization process is less affected by the external environment than those of cross-pollinated plants, making it easier to control extraneous variables during experiments [13]. Most of the genes of wild-type *Arabidopsis thaliana* are highly homozygous, making it easy to induce specific mutations and to precisely construct the corresponding mutants via various physical and chemical methods [14]. Furthermore, as a cruciferous plant, *Arabidopsis thaliana* has a genome of only five chromosomes and approximately 100 million base pairs, which greatly decreases the difficulty and cost of whole-genome sequencing (WGS) [15]. In addition, the WGS of *Arabidopsis thaliana* was completed in 2000 by the Arabidopsis Genome Initiative, popularizing the application of *Arabidopsis thaliana* as an experimental model and the study of functional genes of Arabidopsis during the whole lifecycle of *Arabidopsis thaliana* [16].

In our daily life, we always pay attention to the fruits of plants, such as apples and pears. In botany, a fruit is defined as the specialized seed-bearing structure designed to protect the seeds [17]. The fruit of *Arabidopsis thaliana* is a silique or seedpod that develops from the fertilized gynoecium and dehisces at maturity, allowing the seeds contained inside to spread. Because *Arabidopsis thaliana* is a self-pollinated plant, intrinsic genetic regulatory factors play an irreplaceable role during the fruit development and maturity processes of *Arabidopsis thaliana*. For example, mutations in two functional genes, *KANADI1* and *KANADI2*, directly contribute to the development of the lateral organs (a crucial part of the fruit of *Arabidopsis thaliana*) [18]. Additionally, *CRABS CLAW* (*CRC*) plays a specific role in the gynoecium, the developmental source of the fruit, validating the crucial role of genetic factors in various aspects of fruit development in *Arabidopsis thaliana* [19]. The differentiation of tissues necessary for the opening and dehiscence of the fruit has been considered the core biological process during fruit development [20]. Genes such as *FUL* contribute to the leaf development and meristem identity processes, the phenotype of which has been widely reported in the fruits of *Arabidopsis thaliana* [21]. As we have mentioned above, the genetic contributions to the biological processes of *Arabidopsis thaliana*, especially those of the fruit, have been widely studied and validated, indicating the significance of the identification of the core genes of the physiological processes in *Arabidopsis thaliana*.

Recently, several studies adopted the shortest path algorithm to investigate some important biological and medical problems, such as identifying the genetic determinants in yeast longevity study [22], discovery of novel disease genes [23–28], prediction of novel carcinogenic chemicals [29], *etc*. In this study, using *Arabidopsis thaliana* as a research model as well as validated functional genes, we propose a shortest-path-based method for the prediction of novel genes that contribute to the biological processes related to fruit. A group of genes were accessed by the shortest-path-based method, and several genes were confirmed to contribute to fruit development and maturity through a review of recent publications. The new findings obtained in this study will further promote investigations of the physiological mechanisms underlying fruit development and maturation.

## Materials and Methods

### Materials

#### Validated genes

Validated fruit-associated genes in *Arabidopsis thaliana* were extracted from Plant Ontology (PO, http://www.plantontology.org/download) [30]. First, we downloaded the plant_ontology.obo file from PO (accessed on March 24, 2015), which contained the PO term structure. All of the terms related to fruit and their children terms were considered fruit PO terms, which included fruit (PO:0009001) and its parts (PO:0004707, PO:0008001, PO:0004536, PO:0004535, PO:0008002, PO:0025268, PO:0000033, PO:0008003, PO:0009087, and PO:0009084). This analysis revealed 994 *Arabidopsis thaliana* genes annotated with these PO terms, which are provided in S1 Table and are considered fruit-related genes. The set consisting of these 994 genes was denoted *S*_*v*_.

#### PPIs of *Arabidopsis thaliana*

PPIs play important roles in executing, modulating and maintaining the activities and functions of organisms. Proteins that can interact with each other share common functions [23–26, 31–34]. According to the validated genes mentioned in Section “Validated genes”, we can search for novel fruit-related genes using the PPIs of *Arabidopsis thaliana*. In this study, we adopted the PPIs reported in STRING (http://string-db.org/, version 9.1) [35], which were collected from the following sources: genomic context, high-throughput experiments, (conserved) coexpression, and previous knowledge. The PPIs indicate not only the direct (physical) associations but also the indirect (functional) associations between proteins. To extract the PPIs of *Arabidopsis thaliana*, we downloaded a file called “protein.links.v9.1.txt.gz” from STRING, which contains the PPIs of several organisms, and extracted the lines starting with "3702.". A total of 3,123,482 PPIs of *Arabidopsis thaliana*, which included 25,123 proteins, were obtained. Each of these PPIs contains two proteins, which are represented by their Ensembl IDs, and one score ranging from 150 to 999. This score measures the strength of the interaction: an interaction with a high score indicates that the corresponding proteins have a high probability of interacting with each other. We denoted the score of the interaction between proteins *p*_1_ and *p*_2_ as *Q*(*p*_1_, *p*_2_).

### Network construction

The PPIs of *Arabidopsis thaliana* were used to construct a large network: the 25,123 proteins were defined as the nodes of the network, and the 3,123,482 PPIs were the edges. To further indicate the strength of the interactions in the network, an edge weight was assigned to each edge. The maximum value of the interaction score was 999, and according to the shortest-path algorithm, a low weight suggests a strong association between the endpoints of the corresponding edge. Thus, the weight of edge *e* was defined as follows:

$$w(e)=1000-Q(p_{1},p_{2})$$ (1)

where *p*_1_ and *p*_2_ are the proteins represented by the two endpoints of edge *e*. The information of each edge in the network, including its end-points and its weight, is provided in S2, S3 and S4 Tables.

### Shortest-path-based method

A shortest-path algorithm is a classic graph algorithm that has been widely used in several fields. For example, shortest-path algorithms have been adopted for the discovery of novel disease genes [23–28]. In this study, a shortest-path algorithm was used to discover novel fruit-related genes based on the validated genes mentioned in Section “Validated genes”. The basic theory is that proteins that can interact with each other have common functions [23–26, 31–34]. If one further considers the interaction scores, this fact can be generalized to state that the proteins in a protein-protein interaction with a high score are more likely to share common functions than those in an interaction with a low score. For the network constructed as described in Section “Network construction”, this statement can be converted into the following: nodes (proteins) that are endpoints of an edge assigned a low weight are more likely to share common functions than those that are endpoints of an edge assigned a high weight. Furthermore, if there are several nodes, denoted *n*_1_,*n*_2_,⋯,*n*_*k*_ such that *n*_*i*_ and *n*_*i*+1_ are the endpoints of an edge with a low weight, all of the nodes may share common functions. These nodes may all lie on the shortest path connecting nodes *n*_1_ and *n*_*k*_, and all nodes other than *n*_1_ and *n*_*k*_ may share the functions shared by nodes *n*_1_ and *n*_*k*_. Thus, we searched all shortest paths connecting any two of the validated genes and extracted the nodes included in these paths. Because the endpoints of these paths represent genes in *Arabidopsis thaliana* that are related to the biological processes of fruit, the genes corresponding to the extracted nodes may also be related to these biological processes. The validated genes may also be found as inner nodes in these paths, and these genes were retained to indicate the utility of the shortest-path-based method. The extracted genes that were not validated genes were deemed candidate fruit-related genes in *Arabidopsis thaliana*. In addition, we counted the number of paths containing each extracted gene and termed this value as betweenness, which can indicate the associations between the extracted genes and fruit development in *Arabidopsis thaliana*.

Based on the validated genes, we can identify novel genes in the constructed network. The topological structure of the network is a potential factor that may influence the results because some genes in *Arabidopsis thaliana* can exhibit general associations with other genes, suggesting that the corresponding nodes in the network are general hubs. These genes are more easily identified by the shortest-path algorithm but have less or no association with fruit development in *Arabidopsis thaliana* and should thus be excluded. As a result, a permutation test was performed to exclude these genes. A total of 1,000 gene sets, each with the same size of *S*_*v*_, were randomly produced. For each set, the shortest paths connecting any two genes in the set were searched in the network to determine the betweenness of the genes extracted as described in the above paragraph. Accordingly, 1,000 betweenness values were obtained for the 1,000 randomly produced sets, and one betweenness value was found for *S*_*v*_. The permutation false discovery rate (permutation FDR) was then calculated for each obtained gene as follows:

$$FDR(g)=\frac{M}{1000}$$ (2)

where *M* is the number of betweenness values of randomly produced sets that are greater than that found for *S*_*v*_. A gene with a high permutation FDR exhibits general associations with the 1,000 randomly produced sets and is not specific to the validated gene set *S*_*v*_. Thus, we set 0.05 as the threshold for the permutation FDR to exclude these genes, i.e., genes with permutation FDRs less than 0.05 were selected as the candidate genes.

## Results and Discussion

**Fig 1:**
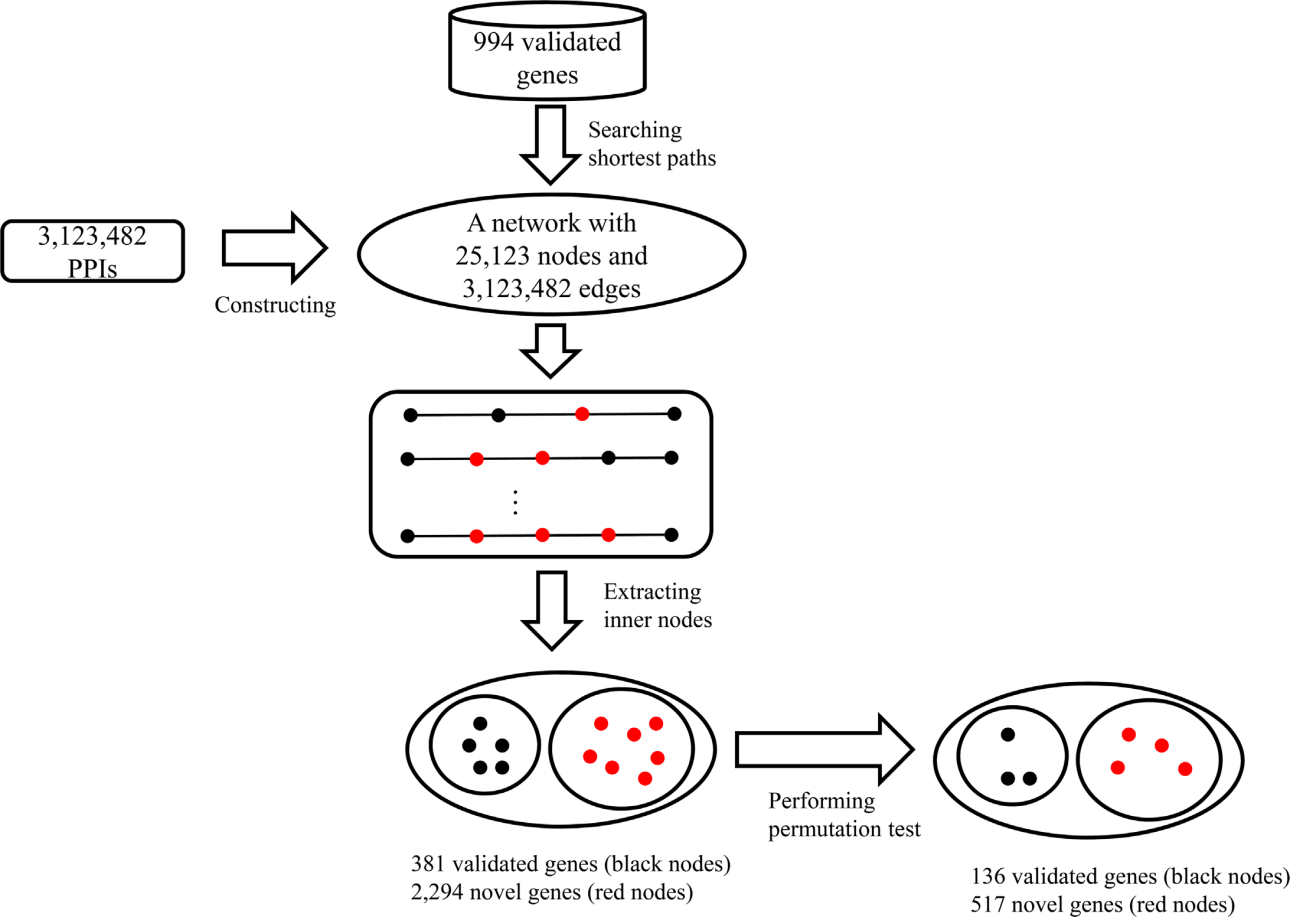
Procedures of and results obtained using the shortest-path-based method for the identification of novel fruit-related genes in *Arabidopsis thaliana*.

In this study, the shortest-path-based method was used to identify novel fruit-related genes in *Arabidopsis thaliana*. The procedure and some of the key results are illustrated in **Fig 1**. This section provides a detailed description of the results and analyses.

### Results of the shortest-path-based method

As mentioned in Section “Shortest-path-based method”, all shortest paths connecting any two genes in *S*_*v*_ were searched in the network. The genes corresponding to the inner nodes in these paths were extracted, and their betweenness was determined. As a result, 3,375 genes were obtained; of these, 381 genes were validated genes, and 2,294 genes were novel genes. Detailed information for these genes is provided in S5 Table.

To control the general genes among the 3,375 genes, a permutation test was performed. We thus calculated the permutation FDR for each gene (provided in S5 Table). After excluding genes with permutation FDRs greater than or equal to 0.05, we identified 653 genes, including 136 validated genes and 517 novel genes, all of which are provided in S6 Table. The 517 novel genes were deemed to exhibit associations with the biological processes of the fruit of *Arabidopsis thaliana* and were considered candidate genes.

### Analysis of the validated and candidate genes

The fruit of *Arabidopsis thaliana* has been widely studied, and the complex regulatory mechanisms underlying its development and maturity have also been extensively studied. In this study, based on the shortest-path-based method, several novel genes were identified and deemed to contribute to the development and maturity of the fruit of *Arabidopsis thaliana*. Furthermore, some validated genes were identified by our method, indicating the robustness of the shortest-path-based method. This section provides a detailed analysis of some representative genes.

#### Analysis of the validated genes

The shortest-path-based method identified 136 validated genes. The five genes with the highest betweenness values were singled out and are listed in **Table 1**. These five genes are also extensively discussed in the following section.

**Table 1:** Five validated genes identified by the shortest-path-based method.

Gene	Also known as	Protein	Function	**Reference**
AT5G63310	ATNDPK2	Arabidopsis nucleoside diphosphate kinase 2	GTP biosynthetic process, response to UV, response to hydrogen peroxide	[38, 39, 75]
AT1G75950	ATSKP1	Arabidopsis Skp1 homologue 1	Core component of the SCF family of E3 ubiquitin ligases, embryogenesis and seedling development	[40, 42]
AT5G57360	ZTL	Clock-associated PAS protein ZTL	SCF-dependent proteasomal ubiquitin-dependent protein catabolic process, circadian rhythm, entrainment of the circadian clock by photoperiod, flower development	[43, 44]
AT1G23190	PGM	Phosphoglucomutase 3	Carbohydrate metabolic process, glucose metabolic process	[45, 46]
AT4G33010	GLDP1	Glycine decarboxylase P-protein 1	Oxidation reduction, glycine catabolic process, response to cadmium ion	[47–49],

**AT5G63310** (encoding Arabidopsis nucleoside diphosphate kinase 2) is a functional gene that contributes to the response to oxidative stress and UV and is involved in phytochrome-mediated light signaling [36–38]. It has been confirmed that UV, as an extrinsic factor, may interfere with the normal development of fruit in *Arabidopsis thaliana* [39]. Because this gene has been found to be expressed in the flower and fruit of *Arabidopsis thaliana*, the responsive and protective function of the gene may contribute to the fruit-associated biological processes in *Arabidopsis thaliana*. The gene ontology analysis of known genes revealed that known fruit-related genes are enriched in specific exogenous stimulus response-associated biological processes, such as **GO: 0006970** (response to osmotic stress) and **GO: 0009416** (response to light stimulus), indicating that the prediction of AT5G63310 is not a special case.

Another gene, **AT1G75950** (encoding Arabidopsis Skp1 homologue 1) is also on the list of known fruit-associated genes, encoding the core component of the E3 ubiquitin ligase and contributes to the auxin-activated signaling pathway [40, 41]. As part of the SCF (Skp1-CUL1-F-box protein) complexes, this gene contributes to embryogenesis and seedling development, which is significant for the early development of *Arabidopsis thaliana* [42]. Another predicted gene, **AT5G57360**, also contributes to the SCF-associated biological processes [43]. Due to its interaction with both CRY1 and phyB, such gene contributes to the regulation of circadian rhythms during the development of the gynoecium, validating its role in fruit development in *Arabidopsis thaliana* [44]. Known fruit-related genes in *Arabidopsis thaliana* are enriched in certain biological processes, including **GO: 0009416** (response to light stimulus), indicating that more functional genes in the list of known genes, such as the two above-mentioned genes, contribute to the development and maturity processes of fruits.

Another gene, **AT1G23190** [also known as phosphoglucomutase 3 (PGM3)], is also on our list of known genes. Such gene not only participates in photosynthesis associated biological processes and glucose metabolic processes as widely reported but according to recent publications, contribute to the development of reproductive tissues, including seeds and fruits [45, 46]. **AT4G33010** [encoding glycine decarboxylase P-protein 2 (GLDP2)] in our prediction list contributes to the dehydrogenation processes of glycine in the fruit [47]. This gene has been reported to contribute to a specific biological process, **GO: 0055114** (oxidation reduction), in which our known genes are enriched, validating its core role during fruit development and maturation [48]. Additionally, as a cadmium-associated gene (**GO: 0046686**), AT4G33010 has been confirmed to contribute to the development and maturation processes of the fruits in various biological systems [49].

#### Analysis of novel genes

In addition to the validated genes, we obtained several novel genes that may contribute to fruit development in *Arabidopsis thaliana*. We then similarly selected the top six novel genes (listed in **Table 2**) with the highest betweenness values and analyzed these genes, as described in the following paragraphs.

**Table 2:** Six candidate genes identified by the shortest-path-based method.

Gene	Also known as	Protein	Function	**Reference**
AT1G09570	FHY2	Far-red elongated hypocotyl 2	Detection of visible light, gravitropism, negative regulation of translation, photomorphogenesis	[50–52]
AT5G59440	ATTMPK	*Arabidopsis thaliana* thymidylate kinase 1	dTDP biosynthetic process, dTTP biosynthetic process, dUDP biosynthetic process	[54]
AT2G18790	PHYB	Phytochrome B	Detection of visible light, entrainment of circadian clock, gravitropism, jasmonic acid-mediated signaling pathway, photomorphogenesis	[51, 53]
AT4G02570	ATCUL1	Auxin-resistant 6	SCF complex assembly, auxin-activated signaling pathway, cell cycle, embryo development ending in seed dormancy	[56, 58]
AT5G20570	ATRBX1	Regulator of cullins1	SCF-dependent proteasomal ubiquitin-dependent protein catabolic process	[58–60]
AT4G29040	RPT2A	Regulatory particle AAA-ATPase 2A	Female gamete generation, leaf morphogenesis	[64]

The first predicted gene, **AT1G09570** (encoding far-red elongated hypocotyl 2), contributes to the regulation of photomorphogenesis [50]. Although no direct evidence confirms that AT1G09570 contributes to fruit development and maturity, the biological process it regulates, photomorphogenesis, plays an irreplaceable role during fruit maturation, implying that AT1G09570 may also contribute to fruit associated biological processes, validating our prediction of such light-detection-associated genes [51, 52]. Similarly to AT1G09570, **AT2G18790** (encoding phytochrome B), which contributes to photomorphogenesis, is also on our list of predicted genes [53]. As mentioned above, photomorphogenesis is crucial for fruit development in *Arabidopsis thaliana*, validating the crucial fruit-related role of this gene [51]. Light is one of the most crucial exogenous stimulants for plants. These two predicted genes contribute to the biological processes associated with photo-stimulation, which can be clustered into the specific biological process of response to endogenous stimulus (**GO: 0009719**), corresponding with the GO enrichment analysis of all of the predicted genes. Considering the validated role of photomorphogenesis in fruit development, our newly predicted gene AT1G09570 and AT2G187790 may definitely contribute to fruit associated signaling pathways. In addition to these two genes, another gene, **AT5G59440** (encoding Arabidopsis thaliana thymidylate kinase), contributes to the TDP/TTP biosynthesis-associated biological processes, particularly during the fruit-associated fertilization processes [54]. This gene has been widely identified in reproduction-associated tissues of *Arabidopsis thaliana*, including the fruit, validating the relationship between this gene and the development and maturity processes of fruit. Validated in rice, barley, maize and *Arabidopsis thaliana*, thymidylate kinase associated genes have been identified in various reproductive tissues, implying their potential functions for fruit development and maturation [55].

The SCF complex, as we mentioned above, contributes to the specific development and maturation processes of the fruit of *Arabidopsis thaliana* [42]. Included in our list of predicted genes, **AT4G02570** (encoding Auxin-resistant 6) contributes to the SCF complex [56]. Considering the role of SCF complex for fruit development and maturation, such gene may further participates in fruit-related regulatory processes [56]. In addition, this gene contributes to the maturation of the reproductive structures and tissues in *Arabidopsis thaliana*, which is also closely related to fruit-associated biological processes, validating our prediction [57]. Additionally, another novel gene on our list of predicted genes, **AT5G20570** (regulator of cullins 1), contributes to the same biological processes as AT4G02570, indicating their similar contribution to fruit development in *Arabidopsis thaliana* [58]. In addition to its participation in the SCF complex, AT5G20570, which is considered a multi-functional gene, also contributes to CUL4-ROC1-DDB1-PRL1 E3 ligase [59, 60]. Such ligase has been reported to interact with DWD proteins which has been confirmed to contribute to the fruit development and formation of specific resistances in various plants including *Arabidopsis thaliana*, validating the irreplaceable role of our predicted novel gene, AT5G20570 for fruit associated biological processes [61, 62]. Another gene, **AT4G29040** (as known as regulatory particle AAA-ATPase 2A), a DNA methylation associated gene, contributes to root meristem maintenance, gametogenesis, and DNA methylation [63]. This gene has been reported to contribute to fruit development by regulating cell division, proliferation and expansion [64]. The gene induces a weak defect in 26S proteasome activity and leads to enlargement of the fruit (increased cell size and decreased cell number), validating the prediction that our screened gene AT4G29040 contributes to fruit development and maturation [64].

### Cross-talk analysis of homological genes in rice and wheat

As we all know, during the evolution, the genome of related organisms (*e*.*g*. animals, plants, *etc*.) share a fair degree of homology, implying that genes identified in one specie (*e*.*g*. Arabidopsis thaliana) may function similarly in other species like rice or wheat. As we all know, not the Arabidopsis thaliana but rice and wheat are two of the major food crops in the world, implying that the identification of reproduction associated genes and pathways may contribute to the genomic modification and further improve the output and quality of such crops. As we have analyzed above, the genes we have identified above may all contribute to reproduction associated pathways (*e*.*g*. fruit fertilization, development and maturation) in Arabidopsis thaliana, suggesting that the homologous genes of such screened genes in rice and wheat may also contribute to fruit associated biological processes and may be one of the core production related regulatory factors. Based on recent publications, we screened out the homologues genes in rice or wheat of our predicted fruit associated genes in Arabidopsis thaliana and validated their underlying functions for reproduction. **AT5G63310**, as we have mentioned above, is a validated fruit associated gene in Arabidopsis thaliana. According to the microarray data of wheat germ extract, the homologous gene of AT5G63310 in wheat, **NDPK2** has been confirmed to express in wheat germ extract and may play a similar role with AT5G63310, validating that the homologue of our screened genes may also contribute to fruit associated pathways in other related plants [65]. Besides the homologous gene of such gene (AT5G63310) in rice, **Os12g0548300**, has also been reported to contribute to fruit development and the formation of specific resistance to the hazardous environment, tightly related to the quality and output of such crop [66, 67]. Apart from such validated genes, the homologue genes of our newly predicted genes have also been identified to be candidate fruit associated genes. Take one of our predicted gene, **AT5G59440**, which is also known as Arabidopsis thaliana thymidylate kinase 1, as an example. Based on current database for rice genome (*e*.*g*. RiceData, RiTE, *etc*.), we identified the homologous gene of AT5G59440 in rice, named as **Os07g44630** [68]. As a member of thymidylate kinase family, such gene has been confirmed to be identified in the bran of rice fruit and play an irreplaceable role in seed development [69]. As for wheat, the family of thymidylate kinase has been confirmed to contribute to the head sprouting process in wheat, implying its specific role in fruit associated biological processes [70]. Wheat blue dwarf (WBD) is a severe disease for wheat, inducing reproductive abnormality [71, 72]. The homologous gene of our predicted gene **AT5G59440**, **TMK**, as a member of the thymidylate kinase family, has been identified to be dysregulated expressed in the abnormal tissues of such disease, validating its potential role for the fruit associated biological processes in wheat [70].

Just like we have analyzed above, some homologous genes in rice and wheat in our screened out genes in Arabidopsis thaliana have been confirmed to contribute to reproductive associated biological processes, according to some recent publications. Such results validates the efficacy and accuracy of our newly presented computational methods and further provide a new research method and perspective into the identification of fruit associated genes in industrial crops.

### Conclusions

This study used a shortest-path-based method for the analysis and prediction of fruit-related genes in *Arabidopsis thaliana*. Based on Plant Ontology database, we identified a group of candidate fruit associated genes in *Arabidopsis thaliana*. Considering the protein-protein interactions, several novel genes were identified using our method, and some of these genes were confirmed to be related to fruit development and maturation. The proposed computational method is a useful tool for the identification of novel genes. Further, comparing with some commercial crops like wheat and rice, our screened out fruit associated genes in *Arabidopsis thaliana* were further validated and showed specific inter-species functional conservation. All in all, the newly found genes yielded our computational methods may provide new insights for the investigation of the fruit of *Arabidopsis thaliana* and certain commercial crops. In this study, we only used protein information to investigate fruit-related genes in *Arabidopsis thaliana* because the protein information is much abundant and complete. In future, some other information, such as microRNA [73, 74], will be considered to add into our method to give a more extensive study.

## Supporting Information

S1 Table 994 validated genes related to fruit of Arabidopsis thaliana. (PDF)

S2 Table Edge information in the constructed network (part one). (TXT)

S3 Table Edge information in the constructed network (part two). (TXT)

S4 Table Edge information in the constructed network (part three). (TXT)

S5 Table Genes identified by the shortest path-based method. (PDF)

S6 Table 653 genes filtering by a permutation test, where 136 are validated genes and 517 are novel genes. (PDF)
