# Supplementary material for: A Shortest-Path-Based Method for the Analysis and Prediction of Fruit-Related Genes in Arabidopsis thaliana
Source: PLoS One. 2016 Jul 19;11(7):e0159519. doi: 10.1371/journal.pone.0159519 (PMC4951011; doi:10.1371/journal.pone.0159519)
Supplement: S1 Table — (PDF) [file pone.0159519.s001.pdf]

S1 Table. 994 validated genes related to fruit of Arabidopsis thaliana

AT4G19440
AT4G32470
AT3G58780
AT2G28000
AT1G75500
AT1G04690
AT5G36700
AT2G21140
AT2G40370
AT3G22690
AT3G19170
AT1G24020
AT4G37610
AT3G01480
AT1G77410
AT5G47760
AT1G11860
AT1G50460
AT1G66730
AT1G05190
AT2G27680
AT3G61415
AT5G61780
AT2G32370
AT4G21650
AT1G78220
AT2G40100
AT5G67360
AT4G26070
AT4G21120
AT5G64560
AT2G45950
AT5G42190
AT1G66340
AT1G21680
AT5G09590
AT1G15390
AT1G30530
AT1G10590
AT4G00360
AT3G01500
AT2G30210

AT1G13930
AT1G73680
AT3G02310
AT2G18550
AT2G45240
AT1G47840
AT2G01520
AT4G33680
AT4G18480
AT3G50820
AT2G36640
AT5G57390
AT4G37680
AT1G29670
AT4G21630
AT5G09410
AT4G38320
AT3G56950
AT3G23980
AT5G56300
AT2G15230
AT5G37830
AT3G52150
AT3G09820
AT5G38940
AT3G04280
AT1G53850
AT5G08100
AT3G54610
AT1G50900
AT2G38080
AT4G32810
AT2G38170
AT3G04680
AT2G05840
AT3G14790
AT4G35830
AT3G10870
AT4G37930
AT3G43810
AT3G02080
AT1G14890
AT2G38470
AT2G37660

AT3G48350
AT3G26650
AT1G15550
AT5G15150
AT2G47510
AT5G25980
AT4G23670
AT1G31630
AT2G30860
AT5G64440
AT5G58230
AT5G36210
AT3G51920
AT2G41100
AT1G56190
AT4G03210
AT5G35630
AT2G25700
AT2G27040
AT5G09690
AT5G11950
AT2G41110
AT5G56640
AT5G10170
AT5G63840
AT5G15450
AT3G20310
AT5G56290
AT4G39650
AT1G78900
AT5G48880
AT1G51680
AT4G15490
AT3G57650
AT3G04580
AT5G53520
AT4G15560
AT3G27000
AT4G28750
AT5G63860
AT5G01500
AT5G27320
AT1G20330
AT2G44750

AT5G03300
AT5G48300
AT5G38110
AT1G08830
AT1G79930
AT3G52420
AT1G31930
AT5G65310
AT1G27050
AT1G16080
AT1G62960
AT5G60760
AT4G16160
AT5G35770
AT2G16600
AT3G01150
AT5G63800
AT5G58330
AT3G07130
AT2G21660
AT1G15080
AT5G62810
AT4G00150
AT3G58970
AT1G04580
AT5G18290
AT1G22270
AT4G25000
AT2G03450
AT4G15500
AT3G20390
AT3G21850
AT5G37180
AT5G06300
AT1G14760
AT1G26830
AT5G54500
AT5G48480
AT1G03130
AT4G28485
AT2G40490
AT3G24170
AT5G51810
AT2G01290

AT5G42180
AT3G21840
AT3G16785
AT3G19700
AT3G06580
AT3G52780
AT1G22450
AT2G45160
AT2G46020
AT4G39210
AT3G52190
AT3G23990
AT2G33150
AT3G22650
AT1G13440
AT3G63150
AT3G51820
AT5G04530
AT1G68560
AT1G10230
AT3G15356
AT2G22430
AT5G60970
AT3G57610
AT1G54360
AT2G06050
AT5G46090
AT1G29920
AT5G04885
AT1G17060
AT5G17920
AT5G53180
AT3G01220
AT1G72320
AT4G03050
AT1G21910
AT4G12560
AT1G30210
AT1G30000
AT1G35730
AT1G20140
AT5G53560
AT5G20950
AT3G28300

AT2G30950
AT2G39730
AT1G36160
AT2G01850
AT5G18380
AT3G28917
AT3G05630
AT1G53070
AT3G44735
AT2G28760
AT3G16470
AT1G14870
AT5G10330
AT5G01040
AT1G05590
AT4G13930
AT1G16010
AT1G53000
AT3G02230
AT1G03900
AT5G01190
AT3G13750
AT1G75020
AT1G63940
AT1G52400
AT2G21170
AT1G08110
AT1G56450
AT2G35960
AT4G33000
AT3G60630
AT3G52720
AT1G73220
AT2G47730
AT5G11450
AT4G33410
AT1G19440
AT1G16470
AT1G77210
AT5G23720
AT3G52930
AT5G14740
AT3G59970
AT2G35980

AT2G46370
AT1G21750
AT2G26710
AT4G13850
AT1G18080
AT2G38110
AT2G28305
AT1G66400
AT3G55200
AT3G43190
AT2G26080
AT5G50380
AT1G75090
AT1G52760
AT4G34060
AT5G13930
AT5G01370
AT3G58700
AT1G22985
AT4G38770
AT4G09650
AT4G27490
AT1G32150
AT5G22640
AT3G25100
AT3G14415
AT4G30830
AT2G05100
AT5G05390
AT2G47050
AT3G12890
AT3G31350
AT1G70730
AT4G27630
AT4G29940
AT3G61440
AT1G17920
AT4G24280
AT5G42740
AT2G25680
AT1G04220
AT5G28500
AT5G04770
AT1G74380

AT3G19270
AT1G67140
AT1G03790
AT2G29090
AT2G22475
AT1G14700
AT3G05420
AT3G26450
AT3G15020
AT5G16020
AT2G29525
AT5G67500
AT2G15090
AT5G02790
AT1G59900
AT1G59610
AT3G08590
AT2G45790
AT5G27630
AT2G16280
AT4G29010
AT1G10170
AT2G39330
AT2G37220
AT3G63010
AT2G21540
AT2G03170
AT3G55990
AT5G43760
AT1G02880
AT3G52890
AT2G46070
AT5G02640
AT1G13020
AT4G34050
AT1G01120
AT4G26200
AT3G04090
AT1G13180
AT5G38420
AT1G06520
AT2G03520
AT1G02930
AT3G10150

AT1G48030
AT2G32440
AT5G38430
AT3G06930
AT2G05990
AT1G19920
AT3G57240
AT3G63140
AT3G47520
AT1G80600
AT1G25450
AT2G36610
AT3G50660
AT5G53490
AT1G71160
AT3G17600
AT5G66510
AT3G01280
AT5G26650
AT4G31300
AT5G07350
AT4G22240
AT3G17390
AT3G18830
AT2G29630
AT1G53240
AT2G45310
AT4G14880
AT5G61960
AT5G01840
AT5G09660
AT3G24280
AT4G34510
AT3G61150
AT1G52030
AT5G24770
AT5G15090
AT5G25350
AT2G44180
AT4G34870
AT5G42980
AT2G03160
AT5G63310
AT2G05710

AT3G20470
AT1G28520
AT4G38970
AT1G27460
AT2G46720
AT4G28520
AT5G48810
AT5G48580
AT5G03790
AT2G36305
AT2G30250
AT1G27080
AT3G08940
AT5G35590
AT5G40370
AT5G66570
AT2G41840
AT1G42970
AT1G31740
AT3G28290
AT3G05120
AT4G28510
AT5G24300
AT1G32060
AT5G63140
AT1G44800
AT1G55250
AT1G16360
AT5G15540
AT5G06250
AT5G57655
AT3G58680
AT4G34580
AT1G04310
AT3G57670
AT2G38230
AT2G06850
AT3G01470
AT1G68530
AT2G37190
AT4G34390
AT2G30870
AT2G32770
AT1G76430

AT1G76450
AT3G56700
AT2G35990
AT1G11720
AT5G13510
AT1G05320
AT3G13470
AT3G09840
AT4G33010
AT1G73250
AT3G58740
AT1G11840
AT3G52300
AT3G62710
AT1G23190
AT1G02205
AT5G26310
AT4G21690
AT1G16280
AT1G47710
AT1G69500
AT5G11670
AT5G19220
AT5G61980
AT5G64570
AT5G49020
AT5G02840
AT5G01180
AT1G77200
AT1G58120
AT3G51860
AT1G80900
AT5G57140
AT1G32470
AT2G40250
AT5G07030
AT3G51160
AT2G31955
AT3G06920
AT3G20500
AT4G25470
AT1G31180
AT2G22760
AT3G17940

AT3G53970
AT1G63690
AT2G20825
AT1G75110
AT4G38740
AT4G28190
AT5G11190
AT2G01170
AT3G60750
AT4G20140
AT3G02150
AT4G34980
AT4G26420
AT3G55510
AT1G73360
AT1G02340
AT4G04640
AT3G48340
AT5G57800
AT1G35720
AT5G14130
AT1G57750
AT4G27780
AT3G48420
AT2G23510
AT3G07650
AT2G20420
AT5G23120
AT1G08200
AT5G26000
AT2G39460
AT1G58180
AT5G53480
AT2G19110
AT1G60860
AT5G07370
AT4G03280
AT5G27450
AT3G54660
AT1G70070
AT4G38510
AT3G55440
AT5G16710
AT5G06950

AT2G31390
AT1G25340
AT5G26594
AT1G75280
AT2G32810
AT4G19230
AT3G03260
AT3G02000
AT3G14420
AT1G67590
AT2G42400
AT1G06680
AT2G28630
AT4G34710
AT3G52810
AT5G39570
AT1G05610
AT5G55480
AT2G45970
AT4G31990
AT1G55600
AT1G50500
AT4G01940
AT1G48850
AT4G26840
AT1G65650
AT5G10730
AT2G33380
AT3G44880
AT4G15570
AT4G14800
AT5G07200
AT2G26640
AT4G18240
AT4G39330
AT2G21330
AT2G03680
AT1G80150
AT1G51980
AT1G76680
AT2G23460
AT5G34850
AT4G24310
AT1G71696

AT5G65800
AT4G01310
AT3G06550
AT1G56340
AT5G38970
AT4G36740
AT3G27670
AT1G67090
AT2G01880
AT5G20270
AT3G61890
AT3G04120
AT5G62790
AT2G31081
AT5G65050
AT5G03630
AT1G10010
AT2G28470
AT1G61720
AT2G19860
AT5G20720
AT4G00416
AT1G18835
AT5G55310
AT1G17260
AT4G33580
AT2G35100
AT5G46880
AT1G78590
AT2G46830
AT3G62100
AT3G27785
AT1G75950
AT1G54990
AT4G21040
AT4G39660
AT4G03430
AT1G77940
AT3G27690
AT2G29130
AT3G20520
AT5G08335
AT4G12250
AT1G78380

AT1G21640
AT1G20020
AT2G24270
AT5G01600
AT1G04420
AT4G24940
AT2G01890
AT5G23260
AT3G12290
AT2G36880
AT4G39260
AT1G17100
AT3G55260
AT5G06320
AT3G17790
AT4G29350
AT5G03260
AT4G09320
AT1G30270
AT5G60540
AT4G19180
AT5G49910
AT3G51550
AT3G25740
AT2G42600
AT4G18390
AT4G21280
AT4G12420
AT3G19640
AT3G59820
AT2G22240
AT1G68010
AT4G24120
AT4G14640
AT2G18915
AT2G35700
AT2G17780
AT2G37270
AT3G18850
AT3G22990
AT2G26670
AT3G45140
AT2G27710
AT3G44110

AT2G23430
AT2G19800
AT1G26210
AT4G13985
AT1G63770
AT1G71100
AT3G59010
AT3G60010
AT1G56550
AT4G12130
AT1G33120
AT1G79280
AT1G10270
AT3G21520
AT2G44080
AT4G11150
AT1G48605
AT5G38410
AT4G09600
AT5G02240
AT1G53510
AT5G44700
AT4G10040
AT1G52940
AT5G20830
AT4G22260
AT1G09210
AT5G50920
AT5G56870
AT2G37940
AT5G07440
AT4G28600
AT2G44950
AT5G20620
AT1G07770
AT1G44090
AT1G32070
AT1G47260
AT5G42020
AT3G52880
AT1G33140
AT3G16950
AT5G54690
AT3G61050

AT5G42810
AT1G07890
AT4G29130
AT5G36790
AT4G21050
AT3G54020
AT5G20940
AT3G53460
AT3G14930
AT2G34500
AT5G45340
AT3G54320
AT5G49570
AT1G03600
AT2G03120
AT4G02280
AT3G19370
AT4G16120
AT1G76690
AT2G39550
AT2G31070
AT1G51590
AT4G13940
AT3G01180
AT2G42620
AT5G14780
AT2G47990
AT2G30330
AT3G63520
AT5G65870
AT5G08070
AT1G76130
AT1G68750
AT3G03250
AT4G18950
AT4G37780
AT4G17460
AT1G02000
AT5G13450
AT5G16290
AT2G38530
AT1G53500
AT1G19570
AT3G25650

AT1G65930
AT3G55330
AT2G13790
AT4G32520
AT2G26890
AT4G26690
AT4G29860
AT3G23150
AT1G05160
AT5G51860
AT5G05340
AT1G11530
AT5G44160
AT2G26250
AT5G11080
AT3G63190
AT3G21160
AT5G63770
AT5G27960
AT1G17110
AT2G37210
AT1G75750
AT4G01060
AT3G17240
AT1G09340
AT1G53310
AT5G08670
AT3G11650
AT2G24640
AT5G65970
AT5G24820
AT1G78570
AT5G49360
AT2G27190
AT5G55160
AT1G15360
AT1G07720
AT2G43070
AT1G72990
AT3G21560
AT5G42100
AT1G22640
AT1G42990
AT5G11320

AT3G02360
AT1G01090
AT5G50600
AT1G02790
AT2G14540
AT3G62030
AT5G20490
AT1G10130
AT1G01290
AT3G23820
AT5G09650
AT5G27540
AT3G16150
AT4G25080
AT5G18170
AT1G27680
AT5G47380
AT2G40790
AT1G31140
AT3G21550
AT3G11630
AT4G11010
AT4G33430
AT3G26790
AT3G52960
AT2G31660
AT5G28540
AT4G30270
AT4G34250
AT3G04790
AT5G23540
AT4G39800
AT5G22830
AT3G59990
AT2G21870
AT3G54890
AT4G26260
AT1G79460
AT5G06610
AT3G15030
AT2G03190
AT1G12310
AT3G20820
AT1G65590

AT5G66190
AT5G01410
AT3G11050
AT3G52380
AT3G53450
AT3G55280
AT3G14940
AT3G54140
AT1G23730
AT3G55800
AT2G34490
AT4G19410
AT5G51480
AT4G31160
AT3G06050
AT3G02730
AT1G03400
AT4G15510
AT4G21080
AT5G63810
AT1G72970
AT5G09360
AT1G64030
AT5G39190
AT5G58070
AT5G24780
AT3G23810
AT1G04950
AT5G42310
AT2G30970
AT1G53230
AT3G07780
AT1G77510
AT3G21830
AT1G10870
AT4G10450
AT4G30850
AT2G46680
AT2G26550
AT1G09780
AT1G68870
AT4G26140
AT2G31690
AT3G46440

AT3G11660
AT2G16500
AT4G10340
AT5G62680
AT1G55480
AT2G20160
AT4G30440
AT3G63490
AT1G09700
AT1G26960
AT4G24250
AT5G60020
AT1G14520
AT1G05230
AT5G59290
AT5G49740
AT3G45280
AT1G42560
AT2G40220
AT3G03980
AT4G05180
AT1G21760
AT1G04080
AT1G69830
AT4G02770
AT1G55350
AT5G18570
AT2G27860
AT2G40940
AT4G03520
AT4G34520
AT4G06746
AT2G46990
AT3G49780
AT5G50260
AT2G44990
AT2G16430
AT2G33800
AT3G23360
AT1G04410
AT5G57360
AT1G70410
AT5G46210
AT3G10920

AT1G74660
AT5G26930
AT4G35190
AT2G01250
AT1G16060
AT2G24200
AT3G10280
AT1G73370
AT1G67830
AT2G36530
AT3G52820
AT1G80330
AT5G18450
AT4G31120
AT4G25480
AT1G01610
AT1G18450
AT2G45220
AT5G02500
AT4G40060
AT5G08680
AT2G27100
AT1G07750
AT5G20650
AT3G26060
AT5G55220
AT5G48160
AT1G65980
AT3G22200
AT4G08920
AT1G76570
AT2G21590
AT1G19150
AT5G05270
AT1G29260
AT2G30370
AT3G52840
AT5G16310
AT5G13980
AT5G67480
AT5G22650
AT3G57870
AT3G55220
AT5G41520

AT5G65020
AT4G34210
AT4G24230
AT1G56380
AT4G24770
AT4G38220
AT1G79550
AT4G04020
AT3G12780
AT5G50400
AT3G11510
AT1G22590
AT1G66970
AT2G41540
AT1G49240
AT2G16730
AT1G52040
AT5G09810
AT3G20040
AT1G73910
AT3G47990
AT5G49190
AT1G64990
AT3G30720
AT4G21030
AT5G55470
AT5G16990
AT5G48100
