# Supplementary material for: A Shortest-Path-Based Method for the Analysis and Prediction of Fruit-Related Genes in Arabidopsis thaliana
Source: PLoS One. 2016 Jul 19;11(7):e0159519. doi: 10.1371/journal.pone.0159519 (PMC4951011; doi:10.1371/journal.pone.0159519)
Supplement: S5 Table — (PDF) [file pone.0159519.s005.pdf]

S5 Table. Genes identified by the shortest path-based method

Ensembl ID	Betweenness	Permutation FDR
*AT5G63310	83568	<0.001
AT1G09570	81073	<0.001
AT5G59440	64367	<0.001
*AT1G75950	62597	<0.001
AT2G18790	62183	<0.001
AT4G02570	61598	<0.001
*AT5G57360	57180	<0.001
*AT1G23190	37375	<0.001
*AT5G20620	36293	<0.001
AT4G29040	33682	<0.001
*AT1G11860	33530	<0.001
*AT2G39730	33121	<0.001
*AT3G03250	32867	<0.001
*AT5G42740	32068	<0.001
*AT3G55800	30025	<0.001
AT5G09900	30006	<0.001
AT2G42790	26876	<0.001
AT3G54050	26451	<0.001
*AT4G38970	23445	<0.001
*AT5G48300	22711	<0.001
*AT5G09660	21995	<0.001
AT5G18200	20382	<0.001
AT2G01570	17034	<0.001
*AT1G79550	16680	<0.001
*AT1G14700	16066	<0.001
AT5G51820	13210	<0.001
*AT1G42970	13104	<0.001
AT1G67070	11838	<0.001
*AT2G45790	10993	<0.001
AT2G39770	10490	<0.001
*AT3G63010	10227	<0.001
*AT2G36530	9898	<0.001
AT2G43010	9400	<0.001
*AT1G15550	9317	<0.001
*AT4G29130	9150	<0.001
AT4G16760	8472	<0.001
AT1G55490	8405	<0.001
AT3G02870	8284	<0.001
AT1G49430	7455	<0.001
AT3G22960	6812	<0.001

AT1G44575	6635	<0.001
*AT3G54660	6577	<0.001
*AT5G20720	6556	<0.001
AT4G26850	6277	<0.001
ATCG00490	5787	<0.001
*AT1G04410	5308	<0.001
AT2G38280	4938	<0.001
AT1G79470	4472	<0.001
AT1G10970	3985	<0.001
AT2G27150	3954	<0.001
*AT3G17790	3765	<0.001
AT1G47220	3754	<0.001
AT1G52410	3717	<0.001
AT1G75080	3580	<0.001
AT5G27380	3222	<0.001
AT1G32340	2958	<0.001
AT2G44160	2322	<0.001
AT1G24470	2083	<0.001
*AT2G01890	1990	<0.001
AT4G25420	1952	<0.001
AT5G60440	1946	<0.001
*AT4G10340	1901	<0.001
AT2G35690	1840	<0.001
AT3G26090	1838	<0.001
AT1G20510	1812	<0.001
*AT5G24300	1802	<0.001
*AT1G13440	1682	<0.001
AT2G02230	1585	<0.001
AT1G13590	1552	<0.001
*AT1G04420	1530	<0.001
AT4G08870	1374	<0.001
AT2G36310	998	<0.001
AT5G01650	988	<0.001
AT2G37020	987	<0.001
AT3G29810	961	<0.001
AT4G20930	911	<0.001
AT5G61130	891	<0.001
AT3G27810	773	<0.001
AT2G07725	729	<0.001
AT5G57410	588	<0.001
*AT3G01500	548	<0.001
AT1G50940	480	<0.001
AT1G13580	445	<0.001

*AT3G53460	311	<0.001
*AT2G21660	297	<0.001
AT3G02040	180	<0.001
AT3G26220	122	<0.001
AT1G77490	118	<0.001
AT5G52920	114	<0.001
AT2G34770	108	<0.001
AT1G75120	87	<0.001
AT5G44640	81	<0.001
AT5G06720	80	<0.001
AT3G13700	67	<0.001
AT4G30920	60	<0.001
*AT5G58330	32	<0.001
AT1G80340	24	<0.001
*AT5G03630	21	<0.001
*AT1G02205	17	<0.001
AT5G06280	14	<0.001
AT5G43300	14	<0.001
*AT1G76430	13	<0.001
AT2G18130	13	<0.001
AT3G48670	12	<0.001
*AT1G52400	9	<0.001
AT4G21000	8	<0.001
AT4G24460	8	<0.001
AT3G15990	6	<0.001
AT2G28210	5	<0.001
*AT1G10010	2	<0.001
AT1G17820	2	<0.001
AT5G64750	2	<0.001
*AT2G40100	1	<0.001
AT1G74458	1	<0.001
AT4G29600	1	<0.001
AT4G37720	1	<0.001
AT5G32475	1	<0.001
AT5G51550	1	<0.001
AT5G61420	1	<0.001
AT1G09530	19548	0.001
*AT4G09320	6259	0.001
AT1G32100	4742	0.001
AT1G20630	4129	0.001
AT5G61790	3866	0.001
AT3G49250	3842	0.001
AT1G22300	3292	0.001

*AT3G09820	3032	0.001
*AT2G45240	2738	0.001
AT2G35635	1891	0.001
AT3G23000	1842	0.001
AT3G25780	1804	0.001
*AT3G62710	1716	0.001
AT2G22860	1491	0.001
AT5G23900	830	0.001
*AT5G17920	640	0.001
AT1G06180	493	0.001
AT4G24160	476	0.001
AT1G24620	18	0.001
*AT5G07200	17	0.001
AT5G10560	13	0.001
*AT5G64570	6	0.001
AT4G23750	4	0.001
*AT1G75110	1	0.001
AT1G10460	1	0.001
AT2G32690	1	0.001
AT2G33510	1	0.001
AT3G28430	1	0.001
AT4G10260	1	0.001
AT4G32480	1	0.001
AT5G58080	1	0.001
AT4G24620	12056	0.002
AT3G20770	6439	0.002
AT4G35090	5729	0.002
*AT3G06580	4276	0.002
AT5G25900	3651	0.002
AT3G23490	3226	0.002
AT4G25100	3027	0.002
AT3G47930	2989	0.002
AT4G17360	1973	0.002
AT2G37260	1608	0.002
*AT3G54890	1523	0.002
AT4G23100	1095	0.002
AT4G38900	973	0.002
AT1G55920	210	0.002
AT5G54650	49	0.002
AT3G42830	10	0.002
AT1G17860	7	0.002
AT2G01735	3	0.002
*AT3G59990	1	0.002

AT5G57540	1	0.002
AT2G36270	9184	0.003
*AT1G06680	2142	0.003
AT5G03730	1975	0.003
*AT4G01060	1135	0.003
*AT5G49360	1069	0.003
AT1G49005	978	0.003
AT3G51590	966	0.003
AT3G59060	640	0.003
AT2G15340	158	0.003
AT3G25760	51	0.003
AT2G47240	35	0.003
AT5G02630	5	0.003
AT3G08770	4	0.003
AT3G16400	3	0.003
AT5G54310	3	0.003
AT3G18180	2	0.003
AT3G28970	2	0.003
AT3G46120	1	0.003
AT3G53550	1	0.003
AT4G18910	1	0.003
AT5G39160	1	0.003
AT3G24650	10673	0.004
AT2G25490	6353	0.004
AT1G67490	4607	0.004
*AT5G01410	2081	0.004
AT1G30620	1910	0.004
*AT3G50820	1876	0.004
AT1G64440	1759	0.004
*AT4G13940	1622	0.004
AT2G18850	990	0.004
AT2G20180	988	0.004
AT4G12920	987	0.004
*AT3G19700	985	0.004
AT1G71270	296	0.004
AT1G02390	4	0.004
*AT4G38320	2	0.004
AT1G69870	2	0.004
AT2G02850	2	0.004
AT1G65730	1	0.004
AT3G03660	1	0.004
AT5G42400	1	0.004
*AT2G33150	23842	0.005

AT3G61140	4897	0.005
AT5G08280	3407	0.005
*AT1G07890	3338	0.005
*AT2G42620	1960	0.005
AT5G26030	1808	0.005
AT4G37040	1081	0.005
AT1G14400	990	0.005
*AT1G72990	918	0.005
AT1G12900	496	0.005
AT1G21560	24	0.005
AT4G04950	16	0.005
AT5G59030	6	0.005
AT2G19500	4	0.005
AT1G29980	3	0.005
AT4G18870	2	0.005
AT1G65090	1	0.005
AT1G80620	1	0.005
AT2G14910	1	0.005
AT2G25150	1	0.005
AT5G15310	1	0.005
AT5G39130	1	0.005
AT1G52340	4070	0.006
AT3G26744	3715	0.006
*AT2G44180	1025	0.006
AT2G28550	742	0.006
AT1G74030	98	0.006
AT3G12460	21	0.006
AT4G09760	9	0.006
AT3G60390	8	0.006
*AT2G32770	6	0.006
AT1G63260	3	0.006
AT4G25900	3	0.006
AT5G57740	1	0.006
AT5G59700	1	0.006
AT2G37040	9204	0.007
*AT3G60750	3654	0.007
AT2G18740	2395	0.007
*AT3G20500	1799	0.007
AT4G25120	1412	0.007
*AT3G04120	1024	0.007
AT4G29640	966	0.007
AT5G44480	848	0.007
*AT1G01610	228	0.007

AT2G31800	37	0.007
*AT5G48580	15	0.007
AT1G78000	14	0.007
AT1G11260	6	0.007
AT2G34130	2	0.007
AT5G25480	2	0.007
AT1G50280	1	0.007
*AT4G33010	36856	0.008
AT4G27430	3670	0.008
AT4G14910	2943	0.008
*AT1G32060	2260	0.008
AT2G34555	988	0.008
AT3G57230	960	0.008
AT3G59380	459	0.008
*AT5G19220	394	0.008
AT5G41000	28	0.008
AT1G15350	1	0.008
AT5G61380	18437	0.009
AT3G13120	9887	0.009
AT4G01690	2804	0.009
AT1G71340	1391	0.009
AT5G58730	1023	0.009
AT4G03510	982	0.009
AT5G22875	533	0.009
*AT1G14870	289	0.009
*AT1G76450	145	0.009
AT1G78050	42	0.009
AT5G04800	5	0.009
AT5G43980	5	0.009
AT1G64405	4	0.009
*AT1G53510	1	0.009
AT1G03475	2681	0.01
*AT4G18480	2249	0.01
AT5G56580	1904	0.01
AT1G20260	1503	0.01
AT1G04300	987	0.01
AT5G67110	980	0.01
*AT2G35980	7	0.01
AT1G19200	3	0.01
AT5G13350	2	0.01
AT5G25250	2	0.01
*AT1G75090	1	0.01
AT1G49190	1	0.01

AT4G09610	1	0.01
AT1G75330	10728	0.011
AT1G69120	8610	0.011
AT5G10480	7316	0.011
AT1G24260	7001	0.011
AT3G29360	4377	0.011
AT2G02500	1763	0.011
*AT1G68560	1265	0.011
AT4G02195	1064	0.011
AT1G79040	365	0.011
AT1G04880	1	0.011
AT5G20570	46365	0.012
AT3G54470	10270	0.012
AT5G35530	7739	0.012
AT3G03780	2030	0.012
AT1G78630	1809	0.012
*AT5G23720	985	0.012
AT3G46580	981	0.012
*AT5G20830	188	0.012
AT1G56430	6	0.012
AT4G12750	1	0.012
AT4G31550	1	0.012
AT2G34590	8952	0.013
*AT1G68530	3070	0.013
*AT4G28750	2239	0.013
AT5G47120	1057	0.013
AT3G03700	985	0.013
AT3G55150	711	0.013
*AT3G15020	314	0.013
AT1G69820	151	0.013
AT3G54830	3	0.013
AT3G03080	1	0.013
AT4G36260	1	0.013
*AT1G32470	9521	0.014
*AT3G26060	2891	0.014
AT1G01040	2062	0.014
AT3G54710	1530	0.014
*AT3G13470	986	0.014
AT3G21070	985	0.014
AT3G22600	131	0.014
AT5G02780	29	0.014
AT5G57720	4	0.014
AT1G17120	1	0.014

AT1G70330	1	0.014
AT5G52390	1	0.014
AT1G76030	9854	0.015
*AT5G63840	3685	0.015
AT1G25350	1235	0.015
AT1G34790	992	0.015
AT1G24510	5	0.015
AT3G01140	1	0.015
AT5G22860	1	0.015
AT1G28300	1264	0.016
AT5G09640	993	0.016
*AT5G42310	32	0.016
*AT5G63140	7	0.016
AT5G10420	2	0.016
*AT4G28190	1	0.016
AT4G24830	11908	0.017
AT3G19820	3607	0.017
*AT2G40220	2871	0.017
*AT1G23730	2516	0.017
AT1G48410	2059	0.017
AT5G04360	981	0.017
*AT2G36305	846	0.017
AT1G09870	8	0.017
AT4G26710	6	0.017
*AT3G52820	1	0.017
AT3G12470	1	0.017
AT3G29320	3567	0.018
AT1G69740	3370	0.018
AT1G26945	988	0.018
AT5G17050	988	0.018
AT3G45070	947	0.018
AT4G12430	11	0.018
AT5G09978	4	0.018
AT5G24900	3	0.018
AT2G28100	2	0.018
AT1G01900	1	0.018
AT5G64860	1964	0.019
AT1G52980	971	0.019
AT2G28190	916	0.019
AT1G15210	5	0.019
AT3G29770	2	0.019
AT4G13770	1	0.019
AT1G21970	2229	0.02

AT5G47700	936	0.02
AT1G08630	155	0.02
AT5G42000	25	0.02
AT1G71300	14	0.02
AT5G51760	9	0.02
AT5G58270	3	0.02
AT5G19690	5576	0.021
AT4G26300	1086	0.021
*AT5G38410	986	0.021
AT1G66280	182	0.021
AT2G25900	9	0.021
AT1G54280	4	0.021
*AT3G46440	1	0.021
*AT5G49910	1	0.021
*AT4G25470	955	0.022
*AT2G46070	925	0.022
AT3G24503	696	0.022
AT5G09420	20	0.022
AT3G55360	5320	0.023
AT3G56940	1990	0.023
AT2G19940	1956	0.023
AT2G38620	1922	0.023
AT2G10940	1243	0.023
AT4G21270	1027	0.023
AT3G48000	988	0.023
AT3G05010	987	0.023
AT1G68650	28	0.023
AT3G49260	2	0.023
*AT1G73360	1	0.023
*AT3G51160	1674	0.024
AT1G01960	1	0.024
AT5G50010	1	0.024
AT1G78300	3719	0.025
AT2G26930	1758	0.025
AT3G54440	1511	0.025
*AT1G05160	23	0.025
AT1G78955	9	0.025
AT2G37060	2	0.025
AT1G74810	1	0.025
AT3G21110	1009	0.026
AT1G04550	971	0.026
AT1G05820	17	0.026
AT2G26680	2	0.026

AT4G08950	2	0.026
*AT3G55510	945	0.027
AT1G05470	322	0.027
AT1G65360	14	0.027
AT1G67940	6	0.027
AT4G22930	7284	0.028
AT2G40030	4770	0.028
AT5G48230	1838	0.028
*AT2G06050	1747	0.028
AT1G01280	968	0.028
*AT2G28000	955	0.028
*AT3G10920	913	0.028
AT5G55250	12	0.028
AT5G28646	4	0.028
AT1G20380	2	0.028
AT4G18395	2	0.028
AT4G33670	2938	0.029
AT4G14700	1891	0.029
AT4G02530	1192	0.029
AT1G14420	620	0.029
AT2G15640	559	0.029
AT3G54210	214	0.029
AT5G17770	158	0.029
AT1G22710	2	0.029
AT3G62980	16158	0.03
*AT3G57610	7842	0.03
AT3G48730	3526	0.03
AT5G17220	2126	0.03
*AT1G09340	1520	0.03
AT2G40380	991	0.03
AT1G42550	985	0.03
AT4G32640	186	0.03
AT1G21230	163	0.03
AT5G02010	8	0.03
*AT1G75750	4	0.03
*AT4G03520	1	0.03
AT5G25390	1	0.03
*AT3G55330	4686	0.031
AT4G28660	1967	0.031
AT1G73720	1010	0.031
AT1G56050	981	0.031
AT1G13370	555	0.031
AT2G20670	15	0.031

AT3G07390	5	0.031
AT4G26320	3	0.031
AT2G02770	1	0.031
AT5G60410	7775	0.032
AT4G03415	976	0.032
AT5G40280	454	0.032
*AT4G23670	372	0.032
AT5G41080	236	0.032
AT5G20250	9	0.032
AT4G26910	6349	0.033
*AT3G23990	1913	0.033
AT1G74210	1375	0.033
AT5G47930	1220	0.033
AT4G32551	1030	0.033
AT4G17870	983	0.033
AT5G20840	968	0.033
AT3G12410	963	0.033
AT4G08770	278	0.033
AT3G63200	35	0.033
AT5G39110	3	0.033
AT2G39450	2	0.033
AT5G19550	28118	0.034
*AT5G42020	3699	0.034
AT5G47030	1517	0.034
AT5G10240	971	0.034
AT2G27030	949	0.034
AT2G30810	260	0.034
AT2G43360	6	0.034
AT4G16690	2	0.034
*AT1G31740	1	0.034
AT1G09790	1	0.034
AT4G19420	1	0.034
AT1G03630	2072	0.035
AT5G66005	1627	0.035
AT4G32140	946	0.035
AT1G09310	855	0.035
*AT4G39260	538	0.035
AT4G16130	516	0.035
AT4G00540	46	0.035
AT3G19050	14	0.035
AT2G26170	3	0.035
AT3G13560	3	0.035
AT3G27580	2	0.035

AT3G04630	1	0.035
AT5G08690	5877	0.036
AT4G08900	4867	0.036
AT4G26900	3504	0.036
AT1G65060	1651	0.036
AT1G32990	1028	0.036
AT5G16760	980	0.036
AT5G66230	942	0.036
AT1G64290	244	0.036
AT2G03500	80	0.036
AT3G60620	34	0.036
AT2G01320	16	0.036
AT1G27950	8	0.036
AT2G16005	4	0.036
AT5G03280	2893	0.037
AT3G57800	973	0.037
AT3G55040	955	0.037
AT1G53750	939	0.037
AT2G42830	185	0.037
AT5G19940	132	0.037
*AT5G11450	101	0.037
AT1G47128	18	0.037
AT5G51750	3	0.037
AT5G03555	988	0.038
AT5G15170	924	0.038
AT4G19490	761	0.038
AT1G25230	633	0.038
AT1G16460	463	0.038
AT2G02860	241	0.038
AT1G21550	24	0.038
AT3G05810	1	0.038
AT3G47450	4249	0.039
AT1G55090	1003	0.039
AT3G01770	170	0.039
AT5G15190	47	0.039
AT5G56360	12	0.039
AT1G15820	6	0.039
AT1G09960	4	0.039
AT1G76590	1	0.039
AT3G48430	2393	0.04
AT4G09000	1777	0.04
AT5G66870	988	0.04
AT3G04720	475	0.04

AT3G14020	124	0.04
AT1G31190	118	0.04
AT2G36580	18	0.04
AT3G24310	17	0.04
AT5G35935	17	0.04
AT1G49670	1	0.04
AT1G65620	2700	0.041
AT3G13440	977	0.041
AT1G80260	976	0.041
AT4G14210	975	0.041
AT2G30000	444	0.041
AT1G24190	364	0.041
AT5G66220	294	0.041
AT4G29340	139	0.041
AT3G44540	6	0.041
*AT2G24200	4	0.041
AT1G19990	2	0.041
*AT1G22640	1	0.041
*AT5G60540	987	0.042
*AT5G57140	900	0.042
*AT1G50900	892	0.042
AT1G48900	860	0.042
*AT5G53560	465	0.042
*AT1G20330	167	0.042
AT3G16370	20	0.042
AT1G11960	18	0.042
AT2G38750	13	0.042
AT2G46650	11	0.042
AT5G05610	1	0.042
*AT4G09650	1852	0.043
AT5G04040	404	0.043
AT4G35020	245	0.043
AT4G39700	186	0.043
AT4G16260	155	0.043
AT4G27440	19	0.043
AT5G64940	1	0.043
AT5G42800	8922	0.044
*AT2G40490	2061	0.044
*AT2G18915	1445	0.044
AT2G45170	907	0.044
*AT1G53500	870	0.044
AT2G27990	774	0.044
*AT2G21330	500	0.044

AT4G00720	346	0.044
AT1G02920	204	0.044
*AT5G14740	129	0.044
AT4G08390	8	0.044
AT2G39750	6	0.044
AT5G51570	4	0.044
AT3G54420	3	0.044
AT1G60110	1	0.044
*AT3G12780	5514	0.045
AT4G35460	106	0.045
AT4G04070	82	0.045
AT4G34260	15	0.045
AT5G08530	6687	0.046
AT4G18960	1969	0.046
AT1G23310	1545	0.046
AT2G37620	957	0.046
AT5G55950	734	0.046
AT1G17070	694	0.046
AT5G20510	344	0.046
AT1G56145	17	0.046
AT5G05690	17	0.046
AT4G24510	9	0.046
AT2G34420	2	0.046
AT3G46110	1	0.046
AT3G59270	1	0.046
AT1G23290	797	0.047
AT3G61260	701	0.047
AT4G17730	622	0.047
AT5G40610	79	0.047
AT1G15910	12	0.047
AT3G25900	10	0.047
AT4G03090	2	0.047
AT2G33040	4057	0.048
AT4G36020	228	0.048
*AT3G45140	91	0.048
AT1G03970	16	0.048
AT4G16370	10	0.048
*AT5G48810	2	0.048
AT1G22500	1	0.048
AT3G54700	1	0.048
AT3G51240	2662	0.049
AT3G20000	2334	0.049
AT2G07050	1931	0.049

AT1G63290	1120	0.049
AT5G14220	997	0.049
AT3G21720	966	0.049
AT2G18770	595	0.049
*AT3G21160	4	0.049
AT5G17520	3	0.049
AT1G48020	2	0.049
AT1G14687	1	0.049
AT1G24520	1	0.049
AT4G28880	1	0.049
AT5G63530	1	0.049
AT2G30490	8865	0.05
AT4G18710	3130	0.05
*AT1G09700	1256	0.05
*AT3G44735	48	0.05
AT3G03000	39	0.05
AT3G49660	7	0.05
*AT5G63770	1950	0.051
AT1G06290	957	0.051
AT5G47110	240	0.051
AT2G35260	163	0.051
AT5G60920	45	0.051
AT1G59740	1	0.051
AT5G23300	6883	0.052
AT2G39760	1945	0.052
AT3G62720	950	0.052
AT5G14420	366	0.052
AT1G49970	2	0.052
AT5G19080	1	0.052
AT3G16770	2928	0.053
*AT1G66340	1992	0.053
AT2G33610	1089	0.053
AT4G22880	1006	0.053
*AT3G01280	144	0.053
AT3G15450	57	0.053
*AT5G65310	6	0.053
AT3G59900	5	0.053
*AT1G52040	1	0.053
AT1G72300	1	0.053
AT2G40435	1	0.053
*AT3G05120	1948	0.054
AT2G36070	1336	0.054
AT2G45820	983	0.054

*AT1G08200	79	0.054
AT5G47840	30	0.054
AT4G27020	17	0.054
AT3G26618	8	0.054
AT3G21580	1	0.054
AT4G35870	1	0.054
AT5G04950	1	0.054
*AT5G66570	1640	0.055
*AT4G31990	29	0.055
*AT1G61720	19	0.055
AT3G16720	17	0.055
AT3G21860	1	0.055
AT5G40810	2387	0.056
AT3G01290	1263	0.056
AT3G07770	465	0.056
AT1G16540	51	0.056
AT1G35140	12	0.056
AT1G53280	1	0.056
AT5G10150	1	0.056
AT3G09150	988	0.057
AT5G20810	289	0.057
AT2G39190	3	0.057
AT1G31850	1	0.057
AT2G39840	1	0.057
AT4G35260	1318	0.058
AT1G14920	1115	0.058
AT2G29650	7	0.058
AT4G20830	3	0.058
AT5G53550	2	0.058
AT5G59880	1	0.058
AT3G17360	1002	0.059
*AT3G21560	989	0.059
*AT3G57650	493	0.059
AT1G71400	1	0.059
AT2G34680	1	0.059
AT3G19920	7	0.06
AT2G44790	2	0.06
*AT4G03280	3517	0.061
AT1G06780	9	0.061
AT1G63970	2596	0.062
AT2G39100	927	0.062
AT2G46880	841	0.062
ATMG00665	827	0.062

AT3G04290	13	0.062
AT5G53890	12	0.062
AT3G51060	3	0.062
*AT2G46990	1	0.062
ATCG00720	7330	0.063
AT5G60600	2769	0.063
AT1G08090	1926	0.063
AT2G19400	972	0.063
AT1G72090	955	0.063
AT5G09930	942	0.063
AT2G46270	17	0.063
AT4G17830	15	0.063
AT2G38540	11	0.063
AT5G64250	6	0.063
AT2G23570	1	0.063
AT5G17320	1	0.063
AT5G26780	2115	0.064
AT1G11755	1316	0.064
AT1G12350	987	0.064
AT3G14130	128	0.064
AT2G26660	2	0.064
AT1G58430	1	0.064
AT4G26120	1	0.064
AT5G13210	1	0.064
AT5G42150	1	0.064
AT5G64800	1	0.064
AT3G51460	4062	0.065
AT2G35530	879	0.065
AT2G19060	3	0.065
AT3G14240	2	0.065
AT5G03910	2	0.065
AT4G33880	1	0.065
ATCG01050	6259	0.066
AT1G65330	3813	0.066
*AT2G40250	1	0.066
*AT3G17940	1	0.066
AT4G02750	1	0.066
AT5G56600	178	0.067
*AT3G61890	2	0.067
AT1G26400	1	0.067
AT2G38710	1	0.067
AT4G18020	1	0.067
AT2G23380	3245	0.068

AT2G01470	1603	0.068
AT4G22080	983	0.068
*AT5G56300	9	0.068
*AT5G65870	3	0.068
AT3G43720	3	0.068
AT4G25450	3	0.068
AT1G58440	1924	0.069
AT1G62330	933	0.069
AT3G18780	452	0.069
*AT4G12420	15	0.069
AT3G56240	7	0.069
AT1G63220	1	0.069
AT1G67530	1	0.069
AT4G32880	1	0.069
AT1G07480	647	0.07
AT3G12500	180	0.07
AT5G19150	32	0.07
*AT3G62030	19	0.07
AT4G21590	10	0.07
AT5G60740	2	0.07
AT1G09010	1	0.07
AT4G24150	1	0.07
AT4G34350	2753	0.071
*AT2G18550	982	0.071
AT2G20370	961	0.071
*AT1G51590	790	0.071
AT4G37520	169	0.071
AT4G09010	168	0.071
AT2G46800	9	0.071
AT4G18790	6	0.071
AT4G36890	6	0.071
*AT2G06850	5	0.071
AT4G00930	1	0.071
AT4G27110	1	0.071
AT5G55240	1	0.071
AT3G21640	1046	0.072
AT1G32780	977	0.072
AT4G35320	602	0.072
AT4G29380	100	0.072
AT1G80760	2	0.072
AT5G36220	2	0.072
AT3G50220	1	0.072
AT1G26110	999	0.073

*AT4G38510	622	0.073
AT4G13020	1	0.073
AT5G61410	1487	0.074
AT5G40770	1083	0.074
AT3G06460	392	0.074
AT2G19740	1	0.074
ATCG00340	2108	0.075
AT5G65430	865	0.075
AT1G24764	4	0.075
AT5G55200	1011	0.076
ATCG00730	974	0.076
AT2G31300	909	0.076
AT4G17600	589	0.076
*AT4G15560	132	0.076
AT4G17340	63	0.076
*AT2G29090	4	0.076
AT3G12740	3	0.076
AT2G23630	2	0.076
AT2G42770	2	0.076
AT2G33330	1	0.076
*AT3G57870	6756	0.077
AT5G15200	2106	0.077
*AT2G05710	1089	0.077
AT1G60990	988	0.077
AT5G09350	527	0.077
AT2G32720	157	0.077
AT3G11710	137	0.077
AT3G01390	119	0.077
AT3G10850	1849	0.078
AT5G67030	1696	0.078
AT1G76490	949	0.078
AT1G09180	618	0.078
AT1G33040	525	0.078
AT4G36810	48	0.078
AT1G73540	1	0.078
*AT1G78900	9063	0.079
AT4G20720	985	0.079
AT4G34200	597	0.079
AT2G18196	26	0.079
AT3G48010	7	0.079
AT2G32950	75389	0.08
AT3G54360	720	0.08
AT5G60620	144	0.08

AT2G33070	6	0.08
AT2G24260	3	0.08
AT5G35735	3	0.08
AT2G37690	420	0.081
AT4G26780	15	0.081
AT1G47600	5	0.081
AT5G58600	4	0.081
AT2G18080	2	0.081
AT1G19970	1	0.081
AT2G03530	1	0.081
AT5G17230	1901	0.082
AT5G55130	1375	0.082
AT5G47810	136	0.082
AT3G03530	48	0.082
AT1G04110	29	0.082
AT5G01360	6	0.082
AT1G16720	1	0.082
AT2G37130	1	0.082
AT5G06570	1	0.082
AT4G09820	8369	0.083
AT3G57050	1872	0.083
*AT3G50660	1465	0.083
AT5G02120	233	0.083
*AT5G05340	1	0.083
AT5G10130	1	0.083
AT5G48670	1	0.083
AT5G51890	1	0.083
AT5G10030	2911	0.084
AT3G55410	2856	0.084
AT5G03290	2623	0.084
AT2G17770	1876	0.084
AT3G23890	990	0.084
AT1G01260	971	0.084
AT3G43440	773	0.084
AT1G48580	26	0.084
*AT3G14940	7	0.084
AT3G19010	7	0.084
*AT4G38770	2	0.084
AT4G02870	1	0.084
AT5G20230	1	0.084
AT3G50060	963	0.085
*AT5G42980	14	0.085
AT4G37270	2	0.085

AT5G04590	2339	0.086
AT4G27060	985	0.086
AT5G14660	181	0.086
AT1G08960	36	0.086
AT4G18280	4	0.086
AT1G76730	2	0.086
AT2G29480	2	0.086
AT2G46870	1	0.086
AT3G07560	942	0.087
AT1G33230	698	0.087
AT3G56650	479	0.087
AT1G09240	235	0.087
AT4G00400	7	0.087
AT1G07745	5	0.087
AT5G42600	1898	0.088
AT5G57160	1555	0.088
AT3G16430	182	0.088
AT4G01660	117	0.088
AT1G04630	106	0.088
*AT5G48100	12	0.088
AT4G08990	5	0.088
AT1G76140	2	0.088
AT1G68875	1	0.088
AT3G18370	1	0.088
AT1G43560	962	0.089
AT2G39290	163	0.089
AT1G63830	1	0.089
AT5G65690	1	0.089
AT1G74260	1309	0.09
AT5G11160	770	0.09
AT4G30210	18	0.09
AT1G30680	6	0.09
AT3G44550	6	0.09
AT1G66850	3	0.09
*AT1G17100	2	0.09
AT2G14170	930	0.091
AT3G59850	5	0.091
AT1G70690	4	0.091
AT2G40300	1	0.091
AT5G21170	2958	0.092
AT5G55810	1987	0.092
*AT5G66190	736	0.092
*AT1G67830	4	0.092

*AT3G13750	3	0.092
AT5G04250	1	0.092
*AT1G48030	20755	0.093
AT5G24520	11030	0.093
AT2G43760	1623	0.093
AT4G36220	885	0.093
AT2G31610	487	0.093
AT4G01320	157	0.093
AT4G31610	4	0.093
AT1G67760	1	0.093
AT4G04880	972	0.094
AT3G01890	877	0.094
AT5G37850	187	0.094
AT2G46240	54	0.094
*AT3G49780	7	0.094
AT4G22820	1	0.094
AT1G31330	4008	0.095
AT3G42850	1655	0.095
AT5G58390	942	0.095
AT1G49530	114	0.095
AT5G15510	8	0.095
*AT1G63690	2	0.095
AT5G09220	1	0.095
AT1G12840	1055	0.096
*AT4G39650	214	0.096
AT4G26610	14	0.096
AT2G22000	1	0.096
AT1G03860	129	0.097
AT5G65940	84	0.097
AT2G25710	9	0.097
AT5G64350	2931	0.098
AT1G65300	2	0.098
AT4G33510	2	0.098
AT2G22420	1	0.098
AT2G21280	984	0.099
AT3G13710	907	0.099
AT5G43060	1	0.099
AT2G17950	6081	0.1
AT5G18690	114	0.1
AT4G35570	53	0.1
AT5G05740	5	0.1
AT1G02050	4	0.1
AT2G01530	2	0.1

AT5G28900	2	0.1
AT1G15750	7341	0.101
AT1G66240	619	0.101
AT2G45470	11	0.101
AT2G31085	9	0.101
AT5G49550	3	0.101
AT5G57590	967	0.102
AT1G34260	609	0.102
AT1G18540	291	0.102
AT5G03530	19	0.102
AT4G26740	15	0.102
AT5G62110	9	0.102
AT2G37550	4	0.102
AT5G14100	1	0.102
AT5G06580	1724	0.103
AT5G40700	24	0.103
AT3G47460	3	0.103
AT1G19660	1	0.103
AT1G68000	1089	0.104
AT2G48070	909	0.104
AT2G45695	54	0.104
AT3G16390	1	0.104
*AT5G13930	6410	0.105
AT1G20050	1136	0.105
AT2G16900	980	0.105
AT5G49650	787	0.105
AT2G30740	254	0.105
AT2G22910	15	0.105
AT5G10300	1	0.105
AT3G18680	11975	0.106
*AT2G03160	1583	0.106
AT3G10370	992	0.106
AT3G14210	6	0.106
AT1G14510	1	0.106
AT1G48150	989	0.107
AT4G04610	239	0.107
AT2G40340	63	0.107
AT2G47470	4	0.107
AT4G22770	3	0.107
*AT1G51680	982	0.108
*AT1G63770	951	0.108
AT3G10550	46	0.108
AT5G20710	7	0.108

AT2G26910	2	0.108
AT2G03780	90	0.109
AT1G03440	65	0.109
AT2G04400	609	0.11
AT5G45670	7	0.11
AT1G70660	2	0.11
AT1G64820	226	0.111
AT4G27410	15	0.111
AT2G25685	4	0.111
AT2G24370	2	0.111
AT2G33830	2	0.111
AT4G29780	986	0.112
AT1G70560	961	0.112
AT2G18290	746	0.112
AT1G65960	607	0.112
AT1G05260	218	0.112
AT5G24410	164	0.112
AT4G04920	29	0.112
AT3G44730	2	0.112
*AT2G22430	17	0.113
AT3G59790	11	0.113
AT5G58650	6	0.113
*AT3G59970	3	0.113
AT3G25040	1	0.113
*AT4G25080	2454	0.114
AT4G23600	123	0.114
AT1G12230	11	0.114
AT3G10740	6	0.114
AT2G44970	3	0.114
AT1G63720	945	0.115
AT5G25420	11	0.115
AT3G61960	2	0.115
*AT3G21830	1	0.115
AT1G49750	1	0.115
AT4G19030	2130	0.116
AT3G18060	1690	0.116
AT3G13930	1375	0.116
AT1G02870	987	0.116
AT5G19820	649	0.116
AT3G27925	13	0.116
AT3G01010	8	0.116
AT5G59430	2	0.116
AT2G42690	1	0.116

AT1G75630	1174	0.117
*AT5G62790	889	0.117
AT1G79850	744	0.117
*AT4G14880	41	0.117
AT2G03220	6	0.117
AT4G35000	515	0.118
AT4G17260	306	0.118
AT4G29210	16	0.118
*AT5G42810	10	0.118
AT3G58750	9	0.118
AT2G05580	3	0.118
AT1G29395	1	0.118
AT3G29800	1422	0.119
AT4G04330	667	0.119
AT3G48930	171	0.119
AT2G29460	40	0.119
*AT2G30950	6	0.119
*AT3G02230	3	0.119
AT5G23320	3	0.119
AT5G46240	1007	0.12
AT5G04330	2	0.12
AT4G20260	1	0.12
AT4G08150	3704	0.121
AT2G26540	674	0.121
AT3G16460	186	0.121
AT5G13300	178	0.121
AT3G51940	4	0.121
AT4G18596	1	0.121
AT1G50420	984	0.122
AT5G20730	962	0.122
AT2G47000	725	0.122
AT4G27160	438	0.122
AT1G75100	25	0.122
AT4G22753	16	0.122
AT2G38940	9	0.122
AT5G38020	6	0.122
AT3G22060	1	0.122
AT3G04520	1128	0.123
AT5G53250	3	0.123
AT1G11190	2	0.123
AT1G70170	1	0.123
AT4G34290	1	0.123
AT1G19670	978	0.124

AT1G73165	329	0.124
*AT2G44080	270	0.124
AT5G43500	74	0.124
AT1G12520	66	0.124
AT2G34860	5	0.124
AT5G18930	2	0.124
AT2G47440	1	0.124
*AT2G46680	4	0.125
*AT4G05180	4	0.125
AT4G27150	2	0.125
AT5G65495	2	0.125
AT5G39760	11	0.126
AT4G01610	1	0.126
AT2G42210	972	0.127
AT2G47190	523	0.127
AT4G39640	79	0.127
AT1G17020	1	0.127
AT1G74890	1	0.127
AT5G46700	1	0.127
AT3G25690	1040	0.128
AT5G15530	816	0.128
AT5G07550	7	0.128
*AT1G73250	1	0.128
AT3G54950	1	0.128
AT5G23010	1711	0.129
*AT4G32470	986	0.129
AT3G27660	8	0.129
AT4G02290	5	0.129
AT2G40690	3	0.129
AT3G27200	2	0.129
AT5G35410	3135	0.13
AT1G19480	409	0.13
*AT1G50500	40	0.13
*AT3G17390	27	0.13
AT1G13270	3	0.13
AT5G13760	1	0.13
AT5G03860	7826	0.131
AT1G08550	985	0.131
AT3G04230	817	0.131
AT5G64120	345	0.131
AT5G66590	15	0.131
AT5G22740	1	0.131
AT1G05900	480	0.132

AT2G05790	10	0.132
AT4G33020	9	0.132
AT4G31150	4	0.132
AT4G22330	442	0.133
AT5G63980	1045	0.134
AT5G15210	61	0.134
AT2G38390	1	0.134
AT4G37830	2787	0.135
AT2G26320	1	0.135
AT2G20890	2324	0.136
*AT1G50460	4	0.136
AT3G51810	1	0.136
AT5G41160	1	0.136
AT3G20330	6889	0.137
AT5G20890	996	0.137
AT3G03190	25	0.137
AT1G51170	3	0.137
AT2G23400	1	0.137
AT1G03430	985	0.138
AT1G25330	982	0.138
AT4G38430	955	0.138
AT3G19510	21	0.138
AT1G08030	5	0.138
AT2G02040	4	0.138
AT3G09440	3	0.138
*AT5G13510	1398	0.139
AT3G15460	962	0.139
AT1G13030	230	0.139
AT1G55880	99	0.139
AT3G12110	35	0.139
AT5G19890	19	0.139
AT1G13610	8	0.139
AT3G12390	2	0.139
AT5G44130	2	0.139
AT1G79450	1	0.139
AT4G35770	20	0.14
AT1G18060	2	0.14
*AT2G46370	873	0.141
AT1G70510	185	0.141
AT4G35450	1367	0.142
AT1G32500	131	0.142
AT1G63280	13	0.142
AT5G46180	600	0.143

AT4G22340	157	0.143
AT4G01190	39	0.143
AT5G45930	37	0.143
AT5G44030	14	0.143
AT1G06550	1	0.143
AT4G12550	1	0.143
AT1G19350	2412	0.144
AT5G15180	47	0.144
AT5G08030	7	0.144
AT5G15860	4	0.144
AT3G53900	899	0.145
AT4G36730	879	0.145
AT1G54220	77	0.145
AT2G19480	1	0.145
AT3G27300	156	0.146
AT1G74470	557	0.147
AT5G63560	528	0.147
AT1G80630	1	0.147
AT2G03760	1	0.147
AT5G26210	1	0.147
AT1G62360	5145	0.148
AT3G46970	3664	0.148
AT1G64670	8	0.148
AT4G10430	4	0.148
AT3G62870	1	0.148
AT2G13360	8434	0.149
AT5G14070	600	0.149
*AT5G57800	525	0.149
AT3G16860	5	0.149
AT3G46520	2343	0.15
AT4G26390	3	0.15
AT5G66280	2	0.15
*AT5G10170	33	0.151
*AT1G58180	12	0.151
AT5G05520	6	0.151
AT1G07030	5	0.151
AT3G58810	2	0.151
AT5G12020	10	0.152
AT2G18690	5	0.152
AT1G25270	2	0.152
AT2G39990	19412	0.153
ATCG00420	5986	0.153
AT5G16440	2753	0.153

AT1G16890	364	0.153
AT1G08510	2	0.153
AT1G43245	1	0.153
AT5G02030	3322	0.154
AT1G70610	60	0.154
AT4G34190	25	0.154
AT5G15630	14	0.154
AT5G41330	4	0.154
AT1G71695	1	0.154
AT3G14740	1	0.154
AT3G20740	8309	0.155
AT5G35790	2164	0.155
AT4G08570	1	0.155
*AT2G31955	248	0.156
AT2G20310	4	0.156
AT1G45249	99	0.157
*AT4G02770	1061	0.158
AT5G49880	1005	0.158
AT3G56310	118	0.158
*AT5G03260	1	0.158
AT5G65720	2167	0.159
AT4G36590	965	0.159
AT5G27600	288	0.159
AT1G75880	2	0.159
AT4G17220	331	0.16
AT1G56650	224	0.16
AT3G56880	2	0.16
AT5G17200	1	0.16
AT2G16370	75271	0.161
AT2G40170	985	0.161
*AT4G21280	359	0.161
AT3G23780	3889	0.162
AT1G15690	1289	0.162
AT4G26600	981	0.162
*AT3G06930	122	0.162
AT2G16660	51	0.162
AT1G03080	5	0.162
AT5G07010	1	0.162
AT3G23050	8950	0.163
AT2G47620	260	0.163
*AT3G04790	5	0.163
AT2G40060	2	0.163
AT1G17880	1	0.163

AT5G52060	1	0.163
AT1G72770	2795	0.164
AT2G40190	2058	0.164
AT4G13510	1145	0.164
AT2G35880	927	0.164
AT2G03590	16	0.164
*AT5G38430	8	0.164
AT2G28850	7	0.164
AT1G55810	1001	0.165
AT3G48030	966	0.165
AT4G35620	40	0.165
AT4G04870	36	0.165
*AT1G77510	6	0.165
AT2G02220	4	0.165
AT3G15730	8658	0.167
AT5G14640	5	0.167
AT2G44350	714	0.168
AT3G58990	17	0.168
AT1G60960	1	0.168
AT5G66720	1	0.168
AT5G50850	5292	0.169
AT2G30170	971	0.169
AT2G22980	1	0.169
AT5G56350	1511	0.17
AT5G49270	3	0.17
AT1G07170	1	0.17
AT2G32930	1	0.17
AT1G15930	1665	0.171
AT1G63050	128	0.171
AT5G66030	99	0.171
AT1G02360	4	0.171
AT2G04750	1	0.171
AT5G65010	25339	0.172
AT5G65930	1679	0.172
AT3G28860	1153	0.172
*AT3G51820	979	0.172
AT1G74590	505	0.172
AT4G33360	412	0.172
AT1G12110	265	0.172
AT1G76710	7	0.172
AT1G54030	3	0.172
AT1G02580	4126	0.173
ATCG00480	86	0.173

AT4G03260	6	0.173
AT2G14210	2	0.173
AT4G05630	1	0.173
AT2G22540	1013	0.174
AT4G13170	301	0.174
AT2G19990	5	0.174
AT4G36250	3	0.174
AT1G48050	1477	0.175
AT5G61580	158	0.175
AT5G11540	13	0.175
AT2G30830	4	0.175
*AT5G37830	3	0.175
AT3G48040	951	0.176
AT2G31650	38	0.176
AT3G25830	5	0.176
AT4G11820	8799	0.177
AT1G62640	5	0.177
AT4G22220	1189	0.178
*AT5G08335	21	0.178
AT3G22230	7	0.178
AT1G51310	6	0.178
AT4G40040	1	0.178
AT2G33290	2273	0.179
AT5G49990	1	0.179
AT5G51560	1	0.179
AT5G56150	525	0.18
AT1G73230	863	0.181
AT3G01850	3	0.181
AT3G08850	1185	0.182
AT1G09080	828	0.182
AT2G26980	105	0.182
AT5G06410	30	0.182
AT3G57550	11	0.182
AT5G10080	1	0.182
AT3G04870	984	0.183
AT4G14680	491	0.183
AT4G15700	202	0.183
AT5G42790	167	0.183
AT3G26740	1	0.183
AT5G55540	575	0.184
AT1G66520	96	0.184
AT3G59530	64	0.184
AT1G55260	37	0.184

AT3G01310	27	0.184
AT1G58290	3462	0.185
AT2G20900	4	0.185
*AT4G34520	2	0.185
AT3G46600	1	0.185
AT1G23710	203	0.186
AT2G45770	70	0.186
AT1G65480	1876	0.187
AT5G43810	67	0.187
AT1G62340	1	0.187
AT4G25890	729	0.188
AT1G01230	448	0.188
*AT3G52890	697	0.189
AT1G02500	6	0.189
AT2G37870	6	0.189
AT3G50530	2	0.189
AT1G22940	673	0.19
*AT1G72970	302	0.19
AT1G09660	24	0.19
AT1G23390	7	0.19
AT1G67990	945	0.191
AT5G44790	156	0.191
AT5G67580	87	0.191
AT5G13790	18	0.191
AT1G51070	1	0.191
AT1G60490	1563	0.192
AT2G38050	978	0.192
*AT3G47520	919	0.192
AT2G24840	58	0.192
AT3G02560	3	0.192
AT4G30190	1083	0.193
AT4G32180	985	0.193
AT5G35550	984	0.193
AT3G61820	113	0.193
AT4G17520	12	0.193
AT3G18730	9	0.193
*AT5G34850	4	0.193
*AT1G71100	1	0.193
AT5G08170	1	0.193
*AT3G55440	10869	0.194
AT1G59820	561	0.194
AT4G23800	122	0.194
AT2G43400	477	0.195

AT1G73160	328	0.195
AT4G25140	21	0.195
AT2G28950	18	0.195
AT4G26510	3	0.195
*AT5G28540	518	0.196
AT2G47940	87	0.196
AT1G78580	20	0.196
AT5G27970	14	0.196
*AT4G00360	3	0.196
AT1G78870	2	0.196
*AT4G37930	87442	0.197
AT5G10760	105	0.197
AT1G24120	72	0.197
AT5G43900	19	0.197
AT5G67130	2	0.197
AT4G26090	1251	0.198
AT3G01435	3	0.198
AT5G61850	14160	0.199
AT2G31490	37	0.199
AT5G57560	5	0.199
AT2G21890	1	0.199
AT5G55730	5	0.2
AT4G32840	393	0.201
AT1G12050	371	0.201
AT1G62510	45	0.201
*AT3G26790	33	0.202
AT4G38660	1	0.202
AT5G16930	1	0.202
AT4G27140	1	0.203
AT1G73590	2213	0.204
AT4G24210	1403	0.204
AT1G04250	1377	0.204
AT5G41410	1349	0.204
*AT3G07780	271	0.204
AT1G02940	8	0.204
AT3G60840	1	0.204
AT3G52140	936	0.205
AT5G58060	91	0.205
AT4G22920	1	0.205
AT2G22040	976	0.206
AT4G13870	963	0.206
AT4G19690	1	0.206
*AT2G38170	1018	0.207

AT2G47170	2	0.207
AT2G48150	2	0.207
AT1G79750	1	0.207
AT4G09800	1191	0.208
AT1G22020	532	0.208
AT3G07270	481	0.208
AT1G79840	104	0.208
*AT1G09210	48	0.208
AT5G47850	8	0.208
AT1G09500	1	0.208
AT4G15770	4663	0.209
AT1G19850	3639	0.209
AT1G72750	936	0.209
AT5G06290	68	0.209
AT2G22780	14424	0.21
*AT3G61440	277	0.21
AT2G21045	3	0.21
AT3G15500	3	0.21
AT1G18400	1	0.21
AT4G23700	1	0.21
AT1G10470	5645	0.211
AT1G08450	292	0.211
AT1G08380	21	0.211
AT5G66150	16	0.211
AT5G24090	3	0.211
AT1G15460	1	0.211
AT1G68060	1	0.211
AT4G35560	1	0.211
AT4G36530	1	0.211
AT1G63020	4352	0.212
AT5G13420	416	0.212
AT4G34900	132	0.212
AT1G54760	18	0.212
AT5G62000	5	0.212
AT4G22200	1005	0.213
AT2G19670	116	0.213
AT1G64350	12	0.213
AT3G45600	10	0.213
AT3G08720	6907	0.214
AT3G15540	4562	0.214
AT1G31360	1	0.214
AT2G20530	40	0.215
AT5G09780	3	0.215

AT3G04060	85	0.216
AT5G47210	6	0.216
AT5G53120	6	0.216
AT2G26560	1	0.216
AT3G09680	1608	0.217
AT3G54220	982	0.217
AT2G18040	979	0.217
AT4G10760	54	0.217
AT2G36060	2	0.217
*AT5G10330	1115	0.218
*AT5G49020	3	0.219
AT1G17190	1	0.219
AT5G07910	58	0.22
AT4G11920	10	0.22
AT3G20580	1	0.22
AT4G26130	1	0.22
AT3G60600	4845	0.221
AT3G19710	1998	0.221
AT5G35360	1002	0.221
AT5G37600	532	0.221
AT3G17820	14011	0.222
AT3G43920	2789	0.222
AT5G41315	2279	0.223
AT1G06430	2	0.223
AT3G17609	877	0.224
AT1G05830	1	0.224
AT5G36880	6632	0.225
AT3G61130	1557	0.225
AT1G23800	281	0.225
AT2G24090	166	0.226
AT1G71200	1	0.226
AT4G35600	1	0.226
AT5G04900	1	0.226
AT3G17760	14	0.227
AT3G56510	3	0.227
AT1G51450	192	0.228
*AT5G01600	70	0.228
*AT2G19800	44	0.228
AT3G05710	7	0.228
AT5G42650	1135	0.229
AT4G29160	977	0.229
AT1G63650	2	0.229
AT2G42160	2	0.229

AT3G57560	3048	0.23
AT2G37630	999	0.23
AT5G05170	21	0.23
*AT5G45340	3	0.23
AT4G05616	1	0.23
AT5G47190	2024	0.231
AT1G61520	1474	0.231
AT5G20630	11	0.231
AT1G63000	8	0.231
AT4G13360	3	0.231
*AT5G46210	38196	0.232
AT5G48840	985	0.232
AT5G24420	974	0.232
*AT1G66970	2	0.232
AT5G09530	123	0.233
AT5G03220	113	0.233
AT1G76600	9	0.233
AT4G00420	6	0.233
AT5G16280	4	0.233
AT3G52940	2621	0.234
AT4G30600	595	0.234
AT3G10670	130	0.234
AT4G02030	16	0.234
*AT5G47760	6	0.234
AT5G07220	1	0.234
AT4G35650	934	0.235
AT3G45610	114	0.235
AT1G30380	21	0.235
AT5G19310	484	0.236
*AT2G39550	64	0.236
AT1G63700	14	0.236
AT2G34250	1	0.236
AT4G20870	32	0.237
AT5G11720	8	0.237
AT2G02480	3	0.237
AT1G52870	2	0.237
AT2G01060	473	0.238
AT1G19910	162	0.238
AT4G13640	4	0.238
AT3G29310	1	0.238
AT2G47030	342	0.239
AT1G61290	81	0.239
AT2G02810	35	0.239

AT1G73965	1	0.239
*AT1G59900	1407	0.24
AT5G39320	945	0.24
AT5G25080	90	0.24
AT4G32150	70	0.24
AT3G01910	42	0.24
AT4G27800	1	0.24
AT4G30840	3428	0.241
*AT3G21840	2	0.241
AT3G17590	1296	0.242
AT3G58460	816	0.242
AT4G17390	7	0.242
AT1G30350	6	0.242
AT4G23590	2	0.242
AT2G34380	1	0.242
AT2G18370	39	0.243
AT2G36910	35	0.243
*AT3G08940	1	0.243
AT1G26100	1	0.243
AT5G01810	5942	0.244
AT5G42080	1193	0.244
AT1G05180	981	0.244
AT3G53740	717	0.244
AT2G43420	41	0.244
AT1G47610	9	0.244
*AT1G02340	987	0.245
AT5G66390	977	0.245
AT3G48360	179	0.245
AT2G30920	1	0.245
AT4G34640	1924	0.246
AT2G19970	7	0.246
AT2G37500	3032	0.247
AT2G21790	1986	0.247
AT3G09260	200	0.247
AT1G33110	50	0.247
*AT1G80600	12	0.247
AT1G80440	11	0.247
AT1G44446	402	0.248
AT1G78480	5	0.248
AT4G00110	1554	0.25
AT3G10800	51	0.25
*AT4G29350	10	0.25
AT1G56330	2069	0.251

AT5G49070	7	0.251
AT5G23670	5	0.251
AT2G28400	2	0.251
AT1G51470	1	0.251
AT1G76160	1	0.251
AT3G22640	1	0.251
AT5G11150	21	0.252
*AT5G15540	2	0.252
AT1G69640	1	0.252
AT4G30580	838	0.253
AT5G60950	2	0.254
AT2G20490	1242	0.255
AT2G30360	1093	0.255
AT2G46560	2	0.256
AT3G56370	27	0.257
AT2G33860	1	0.257
AT5G14380	1	0.257
AT2G28540	2	0.258
AT5G10450	4672	0.259
*AT4G26840	4668	0.259
AT2G35670	358	0.259
AT4G35080	26	0.259
AT5G40420	7	0.259
AT5G49850	1	0.259
AT5G58420	2	0.261
AT4G33770	767	0.262
AT1G07320	169	0.262
AT5G27720	145	0.262
ATCG00020	1606	0.263
AT3G20370	166	0.263
AT2G41460	478	0.264
AT2G01690	294	0.264
AT3G58550	40	0.264
AT2G38760	2	0.264
AT4G26455	2104	0.265
*AT1G53850	197	0.265
AT1G63710	9	0.265
AT5G23240	8	0.265
AT1G15950	2886	0.266
AT2G38120	713	0.266
AT4G04780	422	0.266
AT5G35910	90	0.266
AT1G43850	1022	0.267

*AT3G02080	88	0.267
AT2G27660	1	0.267
AT4G05190	854	0.268
AT2G35040	23686	0.269
AT2G25620	31	0.269
AT1G16900	1556	0.27
AT5G66970	590	0.27
AT4G32410	76	0.27
AT1G64510	43	0.27
AT1G30950	15691	0.271
AT2G27970	664	0.271
AT3G33520	3	0.271
AT5G37780	3	0.271
AT5G10390	2753	0.272
AT4G37560	102	0.272
AT3G13720	1062	0.273
AT3G11750	475	0.273
*AT5G53480	358	0.273
AT4G33930	24	0.274
AT5G51060	2	0.274
*AT3G14415	4872	0.275
AT1G31220	1426	0.275
AT4G35630	596	0.275
AT5G52570	567	0.275
AT5G20980	93	0.275
AT1G63440	31	0.275
AT1G76760	16	0.275
AT2G28790	3	0.275
AT5G05000	1034	0.276
AT4G26080	1018	0.276
AT4G01920	97	0.276
AT1G62980	249	0.277
AT5G16490	3	0.277
*AT5G07370	1	0.277
AT1G62380	938	0.278
AT5G25610	456	0.278
AT5G05660	231	0.278
AT5G64905	1	0.278
*AT3G01180	303	0.279
AT1G15520	3	0.279
AT3G01700	1	0.279
AT3G24225	1255	0.28
AT3G54650	944	0.28

AT3G27380	638	0.281
AT5G23750	1	0.281
AT5G54610	1	0.282
AT2G34650	1293	0.283
AT5G01400	926	0.283
AT1G02640	924	0.283
AT4G18290	23	0.283
AT3G11830	5	0.283
AT3G59760	7	0.284
AT5G32440	7	0.284
AT4G34000	6	0.284
AT5G62360	1	0.284
AT3G03520	114	0.285
AT3G54900	11	0.285
AT2G29490	162	0.286
AT2G40000	22	0.286
AT1G04240	6357	0.287
AT2G15400	999	0.287
AT3G14270	21	0.287
*AT4G02280	3640	0.288
AT4G16720	1009	0.288
AT3G43270	109	0.288
AT1G51110	56	0.29
AT1G35490	6	0.29
AT5G64110	5	0.291
AT1G29150	50720	0.292
AT4G11130	2753	0.292
AT1G55020	157	0.292
AT1G10020	22	0.292
AT1G77450	2	0.292
AT1G75910	1	0.292
AT3G26280	1	0.294
AT1G78770	1044	0.296
AT1G62940	966	0.296
AT3G50360	596	0.296
AT1G06920	81	0.296
AT1G64390	6	0.296
*AT3G51860	3	0.296
AT2G36740	3	0.296
AT1G12240	1846	0.297
AT5G47890	1312	0.297
AT4G16660	891	0.297
*AT3G05420	2	0.298

AT2G47760	2585	0.299
AT3G06940	373	0.299
AT4G24780	23	0.299
*AT1G53240	3	0.299
*AT1G78590	1	0.299
AT3G55560	1	0.299
AT4G11180	1	0.299
AT1G62430	561	0.3
AT3G27830	4	0.3
AT1G66270	1	0.3
AT3G10230	563	0.301
AT1G17840	17	0.301
AT4G14160	1	0.301
AT4G16350	11	0.302
AT1G50030	5554	0.303
AT5G06680	976	0.303
AT1G79940	737	0.304
AT2G42220	2	0.304
AT3G27080	291	0.305
AT4G25700	1	0.306
AT5G14980	2	0.307
AT1G71330	1	0.307
AT2G38700	6491	0.308
AT3G18165	2766	0.308
AT5G49970	1152	0.308
AT5G58140	885	0.308
AT5G44120	53	0.308
AT5G24430	2	0.308
AT2G14750	1329	0.309
AT3G62680	208	0.309
AT2G40430	89	0.309
AT1G09750	1	0.309
AT3G53180	1	0.309
AT5G54840	1	0.309
AT2G39810	22	0.31
AT2G19830	13	0.31
AT5G19520	4	0.31
AT5G52650	26764	0.311
AT5G16750	9231	0.311
AT2G01220	5	0.311
AT1G64280	4895	0.312
AT3G63130	3900	0.312
AT1G24310	324	0.312

AT4G24190	118	0.312
AT4G37000	3	0.312
AT1G11680	3070	0.313
*AT2G39460	697	0.313
AT2G16950	23	0.313
AT5G65683	6	0.313
AT3G50000	1	0.313
AT3G52400	498	0.315
AT2G46410	104	0.315
AT1G16760	3	0.315
AT1G62990	1264	0.316
AT1G12640	1062	0.316
AT1G63120	28	0.316
AT4G25570	6	0.316
AT5G61760	3	0.316
ATCG00280	778	0.317
AT2G23610	6	0.317
AT3G12610	4	0.317
AT4G24440	647	0.318
*AT1G75500	49	0.318
AT1G76810	903	0.319
AT2G22670	5	0.319
AT2G43100	979	0.32
AT5G60390	391	0.32
AT1G70370	3	0.32
AT2G15620	23246	0.321
*AT5G09590	102	0.321
AT3G02470	75	0.321
AT1G62800	557	0.322
AT2G28290	93	0.322
AT1G72610	9	0.322
AT4G09550	1	0.322
AT5G24360	187	0.323
AT3G21060	178	0.323
AT3G23790	84	0.323
AT2G39470	76	0.323
AT1G09415	19	0.323
AT4G36110	44	0.324
AT4G39010	4	0.324
AT4G34850	3	0.324
AT2G30390	631	0.325
AT1G64500	1	0.325
AT2G17500	1	0.325

AT3G51270	473	0.326
AT5G14620	76	0.326
AT4G02500	17	0.326
AT3G61950	2	0.326
AT4G23770	2	0.327
AT1G49620	944	0.328
AT1G01440	142	0.328
AT5G48280	17	0.328
AT4G19170	4	0.328
AT5G16390	4860	0.329
AT5G17420	35	0.329
AT4G00560	5	0.329
AT2G26830	1355	0.33
ATCG00350	204	0.33
AT5G23310	99	0.33
AT1G14880	1	0.33
AT5G47880	809	0.332
AT1G16030	53	0.333
AT3G10140	17	0.333
AT3G22620	1	0.333
AT1G68590	782	0.334
AT3G50740	3	0.334
AT1G78720	1	0.334
AT2G37410	723	0.335
AT3G27060	677	0.335
AT5G03940	325	0.335
*AT1G36160	5447	0.336
AT1G62180	2855	0.336
AT2G18220	1538	0.336
AT2G45810	993	0.336
AT4G26520	141	0.336
AT1G55670	81	0.336
AT1G48520	941	0.337
AT1G26420	4	0.338
AT1G79150	2537	0.339
AT4G40030	854	0.339
AT5G19180	2	0.339
*AT1G26830	4653	0.34
AT4G13710	243	0.34
AT3G14440	5	0.34
AT5G56670	11365	0.341
AT4G00650	986	0.341
AT3G09922	221	0.341

AT5G58690	200	0.341
AT2G45200	24	0.341
AT1G56010	1856	0.342
AT1G48350	1170	0.342
AT3G15950	161	0.342
AT4G36910	6	0.343
AT3G17010	2	0.343
*AT4G01310	5502	0.344
AT1G01580	737	0.344
AT5G55500	12	0.344
*AT1G22450	842	0.345
AT1G06220	510	0.345
AT1G76670	1	0.345
AT5G23190	857	0.346
AT4G28640	12	0.346
AT5G38530	9	0.346
AT5G08620	132	0.347
*AT2G01290	4	0.347
*AT2G34500	3	0.347
AT5G04140	54225	0.348
AT2G26300	7718	0.348
AT5G61010	98	0.348
AT4G01250	1	0.348
AT5G47770	4339	0.349
AT1G32200	60	0.349
AT4G15000	7	0.349
AT3G25070	1245	0.35
AT2G02050	877	0.35
AT4G16155	117	0.35
AT5G20990	103	0.35
AT1G80460	20	0.351
AT5G57050	5450	0.352
AT3G42050	988	0.352
AT2G02390	497	0.352
AT4G16210	154	0.352
AT2G34357	651	0.353
AT5G35170	9	0.353
AT1G18640	620	0.354
AT2G44140	444	0.354
*AT2G03120	25	0.354
AT5G53360	1856	0.355
AT5G08290	3	0.355
AT4G14560	4411	0.356

AT2G27250	4209	0.356
AT5G13630	356	0.356
AT1G59970	16	0.356
AT2G43750	335	0.357
AT3G16920	4	0.357
AT5G15840	678	0.358
AT5G20930	16	0.358
AT1G32440	3	0.358
AT2G48140	1	0.358
AT5G56760	167	0.359
AT3G17840	11	0.359
AT3G01420	8	0.359
AT5G64380	5	0.359
AT1G10500	4	0.359
AT1G49500	621	0.36
AT1G51500	20	0.361
AT1G64090	9	0.361
*AT1G55250	3	0.361
AT2G45890	3	0.361
AT5G51940	53	0.362
AT1G50170	31	0.362
AT3G28500	164	0.363
AT4G02080	1	0.363
AT5G25560	1	0.363
AT1G03090	477	0.364
AT3G45940	23	0.364
AT4G35790	100	0.365
AT3G07050	2	0.365
AT3G25500	31	0.366
AT1G20950	217	0.367
AT4G12440	34	0.367
AT1G79340	2	0.367
AT2G42580	10	0.37
AT5G54770	96	0.371
AT5G05490	28	0.371
*AT1G70410	6	0.371
AT1G52890	3	0.371
AT5G23630	3	0.371
AT1G73820	2	0.371
AT4G37900	3	0.372
AT1G52070	1	0.372
AT1G75680	4	0.373
AT3G06980	3	0.373

AT3G25660	483	0.375
*AT4G16120	1	0.375
AT1G05250	1	0.375
AT1G53400	1	0.375
AT3G16530	1	0.376
AT2G34150	1605	0.377
AT4G01220	5	0.377
*AT2G37190	2	0.377
AT1G08930	1	0.377
AT5G49510	1066	0.378
AT5G49180	233	0.378
AT3G63060	76	0.378
AT1G08710	804	0.379
*AT5G08670	6	0.38
AT3G07960	1	0.38
AT1G60430	911	0.381
AT2G23540	6	0.381
AT2G26690	6	0.381
AT5G22380	1	0.381
AT5G02220	83	0.383
AT3G58730	37	0.383
ATCG00680	1374	0.384
AT2G35370	366	0.385
AT4G38230	2	0.385
AT4G15880	1	0.385
AT2G28670	2	0.386
AT4G01800	1	0.387
*AT4G32520	22160	0.388
AT5G27740	1495	0.388
AT3G26830	446	0.388
*AT1G67090	43	0.388
AT3G17810	28	0.388
AT2G23810	6	0.388
AT1G72440	1965	0.389
AT4G34230	31	0.389
AT4G24970	1	0.389
AT2G22640	1856	0.39
AT2G34630	5	0.39
AT5G12210	2	0.39
AT1G27340	1	0.39
AT4G23710	580	0.391
AT5G01310	1	0.391
AT3G18130	12	0.392

AT1G01050	324	0.393
AT2G10550	162	0.393
*AT3G63140	60	0.394
*AT1G29260	6540	0.395
AT5G62880	14	0.396
AT5G22880	1441	0.397
AT2G03870	1047	0.397
AT3G19280	37	0.397
AT1G05850	4	0.397
AT2G27490	1	0.397
AT5G59820	287	0.398
AT3G52850	105	0.398
*AT3G18850	103	0.398
AT5G44280	1	0.398
*AT3G26650	1110	0.399
AT4G21670	212	0.399
AT5G64200	4	0.399
AT5G09250	3	0.399
AT5G10380	28	0.4
AT5G20060	20	0.4
AT2G07040	1	0.4
AT5G19620	990	0.401
AT2G23150	6	0.401
AT3G26570	977	0.402
AT1G48140	57	0.402
AT5G52840	28	0.402
AT2G44040	989	0.403
AT1G36280	754	0.403
AT3G61650	1	0.403
AT5G13320	1	0.403
AT3G22660	955	0.404
AT2G21160	842	0.404
*AT1G56340	38	0.404
AT5G44750	2	0.405
AT4G09030	1	0.405
AT1G64660	91	0.406
AT3G03960	14	0.406
AT1G03060	1	0.406
AT1G74540	945	0.407
AT2G45290	227	0.407
AT4G27500	94	0.407
*AT1G08110	88	0.407
*AT2G32370	6	0.407

AT4G25730	1625	0.408
AT4G09960	981	0.408
*AT1G05190	136	0.408
AT5G52470	11	0.408
AT5G14030	957	0.409
AT4G34490	17	0.409
AT5G17690	803	0.411
AT5G22310	232	0.411
AT5G11390	6	0.411
AT4G36040	5	0.411
AT1G50430	1100	0.412
*AT2G45220	193	0.412
AT5G24290	152	0.412
AT3G07670	4	0.413
AT1G35710	1	0.413
AT1G22410	159	0.414
AT5G07920	40	0.414
*AT3G01480	29	0.414
AT1G08780	9	0.414
AT4G33500	3	0.414
AT2G39260	2547	0.415
AT5G06000	854	0.415
AT2G32260	4	0.415
*AT2G23430	158	0.416
AT1G16430	18	0.416
*AT2G05840	37	0.417
AT5G42420	10	0.417
AT3G54920	4	0.417
AT1G77090	2	0.417
AT3G06340	6	0.418
*AT1G65590	3	0.418
AT3G18290	3	0.418
AT5G22330	978	0.42
AT4G16780	2	0.42
AT5G40850	31	0.421
ATCG00270	204	0.422
AT1G16590	19	0.423
AT5G23710	1051	0.424
AT1G13890	465	0.424
AT1G32130	4	0.424
AT2G04030	2	0.424
AT3G59400	206	0.425
AT3G61870	51	0.425

AT2G27960	1919	0.426
AT5G08300	638	0.426
AT4G31400	201	0.426
AT1G30860	25	0.426
AT5G19530	70	0.428
AT4G14550	11	0.428
AT1G31780	221	0.429
AT1G77720	765	0.43
AT3G52560	362	0.43
*AT5G18380	49	0.43
AT2G37990	12	0.43
AT1G50250	10	0.431
AT5G35460	1	0.431
AT1G52560	24	0.432
AT5G21274	287	0.433
AT3G25140	26	0.434
AT2G15890	2	0.434
AT2G44610	1	0.434
AT3G07040	1118	0.435
*AT2G20420	1054	0.435
AT1G02280	106	0.435
AT5G56720	3	0.435
AT3G05000	3	0.436
AT5G09520	108	0.437
AT5G09450	8	0.437
AT1G31480	1	0.437
AT5G41040	2	0.439
AT5G11690	202	0.44
AT1G18250	20	0.44
AT1G51800	3	0.44
AT4G16700	927	0.441
AT5G23290	174	0.441
*AT3G02730	6	0.442
AT1G62750	4	0.442
AT5G43350	2	0.442
*AT5G36790	1385	0.443
AT3G57140	793	0.443
*AT5G09810	23	0.444
AT5G58890	7	0.444
AT3G61530	679	0.445
AT1G68920	2	0.445
AT3G51770	2586	0.446
ATCG00150	77	0.446

AT5G67630	52	0.446
AT3G13180	27	0.446
AT4G37910	9	0.446
AT3G24320	2	0.446
*AT5G06950	1025	0.447
AT1G17340	281	0.447
AT5G15490	113	0.447
AT3G55610	13	0.447
AT5G58220	1266	0.448
AT4G11600	2	0.448
AT4G21540	429	0.449
AT5G18070	89	0.449
AT5G57220	3	0.449
AT4G03240	1493	0.45
AT5G60670	301	0.451
AT2G33210	9	0.451
AT4G01150	2	0.451
*AT5G55480	1	0.451
AT1G48160	48	0.452
AT5G64330	883	0.453
AT1G74360	5	0.453
AT1G69770	285	0.455
AT2G02710	231	0.455
AT1G63680	3	0.455
AT1G09070	1	0.455
AT2G01350	1240	0.457
AT1G22310	6	0.457
AT2G46700	4	0.457
AT3G21690	10	0.458
*AT3G55260	9	0.458
AT4G37760	2	0.458
AT2G38440	911	0.459
AT5G14530	152	0.46
AT5G63670	611	0.461
AT3G13320	1	0.461
AT1G73050	15	0.462
AT2G20000	8	0.462
AT5G56030	131	0.463
AT5G51910	6	0.463
AT5G60170	2	0.463
AT2G28160	649	0.464
AT3G09560	80	0.464
AT3G61220	6	0.464

AT1G14910	4	0.464
*AT2G01250	2	0.464
AT5G48600	30	0.465
AT5G27030	5	0.465
AT3G59410	942	0.466
AT2G18250	1	0.466
AT2G24540	773	0.467
AT2G01950	26	0.467
*AT1G47710	10	0.467
AT3G25150	4	0.467
AT1G49770	1	0.467
AT5G66680	5524	0.468
AT4G21530	565	0.47
AT3G62310	182	0.47
AT5G49720	62	0.47
AT4G22800	621	0.471
AT5G66120	161	0.471
AT1G08490	218	0.472
AT3G62550	5	0.473
AT1G69390	50	0.474
AT5G05620	2	0.474
*AT1G01290	1	0.474
AT5G48870	131	0.475
AT2G30070	964	0.476
AT4G33240	108	0.476
AT2G46480	14	0.476
AT1G69600	206	0.477
AT4G34160	83	0.478
AT5G02440	1	0.478
AT5G62410	18	0.48
AT2G22230	1	0.48
AT3G02210	1	0.48
AT4G39890	1	0.48
AT2G32590	13	0.481
AT1G54970	3	0.481
AT5G42950	3	0.481
AT3G19450	2	0.481
AT5G03760	1	0.481
AT1G01060	2287	0.482
AT2G27050	1	0.482
AT2G05520	1015	0.483
AT3G02780	26	0.483
AT1G65440	4	0.483

AT5G11880	3	0.483
AT5G38460	1025	0.484
AT2G45670	58	0.484
AT5G51200	20	0.484
AT5G08650	13	0.484
AT5G57890	763	0.485
AT5G13080	912	0.486
AT2G41880	71	0.487
AT1G16780	7	0.488
AT1G07810	1	0.488
AT3G62600	8	0.489
AT1G32330	1	0.489
AT5G02870	312	0.49
AT4G11260	1886	0.492
AT1G04730	169	0.492
AT5G55590	1	0.492
AT4G00290	4	0.494
AT3G20920	669	0.495
AT1G11890	80	0.497
AT1G75930	2	0.497
AT4G24700	2	0.497
ATMG00070	827	0.498
AT3G55400	29	0.498
AT2G18960	24	0.498
AT2G37180	4	0.498
AT3G05760	57	0.499
AT1G07420	45	0.499
AT5G12200	26	0.499
AT4G37870	716	0.5
AT4G37670	410	0.5
AT3G44890	198	0.5
AT4G16143	363	0.502
AT4G38240	42	0.502
*AT5G35630	46473	0.504
AT1G74960	1971	0.504
AT5G24270	49	0.504
AT3G10390	18	0.504
AT1G78510	227	0.505
AT5G65410	4	0.505
AT4G13610	4	0.506
AT2G46820	8	0.507
AT1G79650	1954	0.508
*AT1G64990	5	0.508

AT2G47060	3	0.508
AT1G37130	17887	0.509
AT3G58660	61	0.509
AT3G11980	6	0.509
AT5G02410	1020	0.51
AT5G36160	70	0.51
AT4G14147	6	0.51
AT2G38670	1357	0.511
AT3G47640	249	0.511
AT2G21210	1	0.511
AT5G58670	3635	0.512
*AT4G28510	1	0.512
AT5G49460	75	0.513
AT4G07523	2	0.513
AT4G26110	296	0.514
*AT5G09650	155	0.515
AT2G41450	1	0.515
AT1G20575	2142	0.516
AT1G77760	803	0.516
AT1G30110	4	0.516
AT2G46280	25899	0.517
AT2G27170	822	0.517
AT1G75010	49	0.517
AT2G19080	9	0.517
AT4G01480	25	0.518
AT1G18590	83	0.519
AT3G44830	72	0.519
AT1G14350	15	0.519
*AT2G41100	9	0.519
AT1G27980	13	0.521
AT5G64370	13	0.521
AT1G42440	473	0.522
AT4G18780	14	0.522
AT1G56170	3	0.522
AT5G44420	6401	0.523
AT5G64070	3541	0.523
ATCG00710	1917	0.523
*AT5G49570	970	0.524
AT1G51950	1	0.524
AT3G49960	1	0.524
AT1G21980	3382	0.525
AT5G64000	18	0.526
AT3G50110	140	0.527

AT1G80500	2	0.527
*AT5G49190	3	0.529
AT5G44560	3	0.529
AT2G44660	1020	0.53
AT1G67730	390	0.53
AT1G19700	81	0.53
AT2G36990	6	0.53
AT2G40550	281	0.531
AT2G19450	82	0.532
AT1G74320	4	0.532
AT1G04210	113	0.533
AT5G50470	2	0.533
AT2G35020	1	0.533
AT3G13550	118782	0.534
AT2G26760	14	0.534
AT3G04640	4	0.534
AT4G33950	4855	0.536
AT3G60880	985	0.536
AT2G38770	3	0.536
AT1G80080	15	0.537
AT5G37055	6	0.537
AT2G25060	2	0.537
AT4G16190	2	0.537
AT1G06590	574	0.538
*AT3G24170	50	0.538
AT3G10340	12	0.538
*AT1G05590	6	0.538
AT1G27390	3	0.538
AT1G04640	1	0.538
AT2G23310	1	0.538
AT5G46840	387	0.539
AT2G27340	4	0.539
AT4G12730	4	0.54
AT2G41620	291	0.541
AT1G64150	28	0.541
AT4G39670	1	0.541
AT1G55900	3594	0.542
AT3G27740	23	0.543
AT5G04510	22	0.543
AT4G29330	6	0.543
AT3G54690	985	0.544
AT2G39960	163	0.544
AT4G04955	140	0.544

AT3G47220	5	0.544
AT3G21350	1673	0.545
AT5G02310	9	0.545
*AT1G20020	13	0.546
AT3G44590	4239	0.547
AT3G04770	1195	0.549
AT1G12920	608	0.549
AT4G39350	2	0.549
AT2G01140	3	0.551
AT3G12590	1	0.551
AT5G03030	1	0.551
AT3G12280	9161	0.552
AT3G59030	3	0.552
AT1G06390	1	0.552
*AT4G25480	100	0.553
AT3G01160	1	0.555
AT5G53530	10	0.557
AT4G33150	675	0.558
AT5G58040	129	0.559
*AT1G21750	18	0.559
AT3G14310	5	0.559
AT3G23060	3	0.559
AT5G17290	1	0.56
AT4G32915	101	0.561
AT1G77300	53	0.561
AT4G10710	52	0.562
AT1G12960	16	0.563
AT2G38650	6	0.563
AT5G17410	3	0.563
AT1G75510	1095	0.564
AT1G18870	2	0.565
AT1G69840	5	0.566
AT3G63080	1	0.566
AT5G39510	622	0.567
AT2G28520	220	0.569
AT4G38080	11	0.57
AT4G26500	12	0.572
AT3G05040	16	0.573
AT2G03750	15	0.573
AT3G20050	2022	0.574
AT5G06140	1002	0.574
AT4G17680	103	0.574
AT3G54170	3	0.574

*AT4G30440	1041	0.575
*AT3G02360	11	0.575
AT2G18330	6	0.576
AT3G60820	6	0.576
AT1G54830	127	0.578
AT3G23250	12	0.58
AT1G79920	2	0.58
AT2G31880	2	0.58
AT4G10960	36	0.581
AT2G43030	3177	0.582
AT1G14290	435	0.582
AT2G47630	9	0.582
AT5G58260	181	0.583
AT4G34740	137	0.583
AT5G35750	92	0.583
AT1G76630	4	0.583
AT3G52090	1941	0.584
AT1G05510	7	0.584
AT4G15530	39	0.585
*AT1G79930	8	0.586
AT3G01120	23	0.587
AT1G73190	12	0.587
AT3G11520	969	0.588
AT1G18360	23	0.589
AT3G15580	10	0.589
AT3G50500	9	0.59
AT5G38640	1	0.59
AT1G34430	4736	0.592
AT1G74100	825	0.592
AT5G09310	3	0.592
AT5G14240	2	0.592
AT4G24570	351	0.593
AT3G15710	59	0.593
AT3G46960	3	0.594
AT1G75660	1	0.594
AT3G03050	1	0.594
AT1G48130	460	0.596
AT1G53165	2	0.596
AT4G04740	975	0.598
AT4G13210	16	0.598
AT3G12810	6	0.599
AT3G06350	180	0.601
AT3G55190	173	0.601

AT2G16960	4	0.601
AT5G16120	119	0.602
AT1G72880	932	0.605
AT5G38900	2	0.605
AT1G54210	1	0.605
AT1G56070	375	0.607
AT3G10690	58	0.607
*AT1G01090	2128	0.608
AT1G03330	1115	0.608
AT2G25610	1082	0.608
AT5G63870	284	0.608
AT3G29270	95	0.608
AT3G12930	5	0.608
*AT3G48420	811	0.609
AT5G63990	10	0.609
AT5G08410	1	0.609
AT1G16970	3052	0.61
AT4G39940	78	0.61
AT2G29420	2	0.61
AT1G54250	2	0.612
AT2G16530	2	0.612
AT3G11430	117	0.613
*AT1G13180	15	0.613
AT4G31700	8830	0.614
AT3G10260	49	0.614
AT1G48830	1451	0.615
AT5G05190	5	0.615
AT2G35650	1	0.616
AT5G10360	516	0.617
AT2G15790	31	0.617
AT3G52590	5085	0.618
AT1G60970	965	0.618
*AT1G22270	109	0.619
AT1G21250	82	0.619
AT3G13870	1	0.619
AT4G38580	191	0.62
AT4G26530	3	0.621
AT1G24100	828	0.622
AT5G09790	185	0.623
AT2G18710	91	0.623
AT5G57090	910	0.624
AT2G23420	15	0.624
AT1G19180	4940	0.625

AT5G16690	20	0.625
AT3G23940	3168	0.626
AT1G51710	740	0.626
AT5G27150	43	0.626
AT1G17160	4	0.626
AT3G24090	125	0.627
AT2G31260	39	0.628
AT3G51520	74	0.629
AT1G31870	4	0.629
AT5G14910	1	0.629
AT2G43560	645	0.631
AT1G08700	2	0.631
AT2G39400	1	0.631
AT4G01900	1145	0.632
AT5G39340	1067	0.633
AT1G27730	624	0.633
*AT4G34210	69	0.633
AT2G31340	1	0.633
AT2G44810	45	0.634
AT1G73480	1	0.634
AT4G16800	567	0.635
*AT3G11510	1	0.635
AT1G80940	2	0.636
AT1G74090	27	0.637
AT4G02620	819	0.638
*AT4G27780	185	0.638
AT5G13710	20	0.638
AT1G18890	4	0.64
AT1G13420	1	0.641
AT2G18700	4	0.642
AT3G57080	2	0.642
AT2G24850	1	0.642
AT4G34570	1081	0.643
AT2G11810	161	0.643
AT5G15770	88	0.644
AT3G03600	5	0.644
AT3G62230	1	0.644
AT4G33905	1	0.644
AT5G19910	3	0.645
AT4G16420	1469	0.646
AT3G20350	32	0.646
AT3G48560	1352	0.648
AT1G10070	3622	0.649

AT1G65030	105	0.649
AT5G35520	17	0.649
AT1G80920	10	0.649
AT3G16857	1051	0.65
AT2G35940	118	0.65
AT2G19240	1	0.65
AT2G33450	229	0.651
AT1G68050	175	0.651
AT3G54670	789	0.652
AT4G30510	24	0.652
AT2G20360	5103	0.653
AT3G25250	47	0.653
*AT1G55480	24	0.653
AT3G06060	20	0.653
AT5G47670	18	0.653
AT3G49680	20	0.654
AT3G14080	4	0.654
AT5G53460	5806	0.655
AT3G02580	720	0.657
AT2G30200	1973	0.659
AT2G21390	5	0.659
AT4G17615	12635	0.66
AT4G34030	981	0.66
AT5G03520	54	0.66
AT1G69190	475	0.661
AT1G22920	3789	0.662
AT5G11260	1864	0.662
AT1G12250	1	0.662
AT1G52240	407	0.663
AT5G03850	68	0.663
AT5G51120	33	0.664
*AT5G55220	1328	0.665
AT5G50210	1304	0.665
AT2G37560	784	0.665
AT3G56800	4232	0.667
AT5G62300	332	0.667
AT3G20550	5	0.668
AT2G02990	1	0.668
AT2G43900	1	0.668
AT2G42540	735	0.669
AT5G46330	1056	0.671
AT5G17330	123	0.671
AT2G07340	17	0.671

AT2G01760	11	0.671
AT5G55190	9	0.671
AT5G01150	485	0.673
AT4G12800	55	0.673
AT1G09840	1	0.673
AT1G30120	1056	0.674
AT2G29570	445	0.674
AT1G77740	54	0.674
AT3G24140	15	0.674
AT3G59810	4	0.674
*AT5G07440	3	0.675
AT5G47780	6	0.677
AT1G14150	252	0.678
AT5G67400	206	0.678
AT2G22450	3	0.678
AT1G10520	86	0.679
AT3G25920	23	0.679
AT1G04680	8	0.679
AT1G70310	1	0.679
AT2G47450	1182	0.68
AT1G11090	36	0.68
AT2G20610	831	0.681
AT5G52310	672	0.681
AT3G12050	132	0.681
AT4G38800	91	0.681
*AT1G47260	4232	0.683
AT1G21400	3617	0.683
AT5G42480	49	0.683
AT1G73030	8	0.683
*AT2G21170	501	0.684
AT1G20440	6	0.684
AT3G49500	755	0.686
AT3G09940	240	0.686
AT1G30460	96	0.686
AT2G45760	47	0.686
AT4G02930	1	0.686
AT4G32040	118	0.687
AT1G27480	3	0.687
AT4G20960	1	0.687
*AT1G56450	1107	0.688
AT1G74310	48	0.688
AT3G57510	2	0.688
AT3G23700	600	0.689

AT1G18040	1656	0.69
AT2G27600	977	0.691
AT5G13840	1423	0.692
AT1G09830	137	0.692
AT4G39030	1	0.692
AT1G03360	987	0.693
AT5G41480	475	0.693
AT1G20090	268	0.694
AT1G09770	2274	0.695
*AT1G02790	46	0.695
AT1G02730	9	0.696
AT4G38600	977	0.697
AT1G33270	26	0.697
AT3G45980	61	0.698
AT3G57490	42	0.698
AT3G44300	2	0.698
*AT4G33430	2076	0.699
AT5G63650	13	0.699
AT3G15940	3	0.699
AT1G24140	1	0.699
AT3G51840	38	0.7
AT5G55280	11	0.7
AT5G22410	2	0.7
AT3G47810	124	0.701
AT2G34710	10	0.701
AT4G25490	730	0.702
AT1G15570	715	0.702
*AT4G04640	423	0.702
AT3G11730	48	0.702
AT2G17630	25	0.702
AT1G64355	70	0.703
AT1G46408	34	0.703
AT5G43690	2	0.703
AT1G55750	6929	0.704
AT3G51310	9	0.704
AT3G51280	1	0.704
AT5G46110	38	0.705
AT1G76040	2	0.705
AT5G45970	2	0.705
AT5G10140	3466	0.706
AT5G20920	145	0.706
AT1G79590	4	0.706
AT3G45780	883	0.707

AT5G14170	3	0.708
*AT3G02150	706	0.709
*AT5G54690	195	0.709
AT4G39370	1	0.709
AT1G02690	510	0.71
AT4G05420	42945	0.711
ATCG00670	1687	0.711
AT3G28715	127	0.711
AT1G29900	58	0.711
AT3G10380	32	0.711
*AT4G19410	4	0.711
AT4G02580	6170	0.712
AT1G53900	485	0.714
AT3G48570	73	0.714
AT1G14810	975	0.715
AT2G26800	567	0.715
AT5G04600	524	0.716
AT2G41630	311	0.716
AT4G23650	8	0.717
AT5G65360	41	0.719
AT1G72040	20	0.721
AT2G41680	3	0.721
AT2G02950	883	0.722
AT5G61170	686	0.722
*AT3G14930	1	0.722
*AT5G41520	7239	0.723
AT2G41490	2	0.726
AT5G63680	3	0.729
AT3G05210	1092	0.73
AT4G30320	14	0.73
AT3G59280	1	0.73
AT1G08190	81	0.731
*AT3G52930	5	0.731
AT3G10640	1	0.731
AT1G31230	952	0.734
AT5G16620	987	0.736
AT3G53260	40	0.736
AT1G18730	20	0.736
AT1G26840	5	0.736
AT4G00020	2904	0.737
AT5G13570	1714	0.737
AT5G07630	3	0.738
AT2G22490	173	0.739

AT1G06950	987	0.74
AT1G16570	1	0.74
AT2G24820	3	0.741
AT3G49160	3	0.741
AT5G07110	3	0.741
AT1G64520	708	0.742
AT5G47720	42	0.743
AT1G75850	23	0.745
AT1G54100	2634	0.746
AT1G15310	738	0.746
AT3G46740	93	0.746
AT3G29090	854	0.747
AT1G14345	50	0.747
AT1G35680	461	0.748
AT5G24160	6	0.749
AT3G45300	881	0.751
AT4G33050	3	0.751
AT1G30520	1	0.751
AT4G19640	75	0.752
AT5G01820	11139	0.753
AT1G80420	1	0.753
AT5G51700	1200	0.754
AT5G61900	80	0.754
AT2G20060	40	0.755
AT4G21070	1	0.755
AT2G42710	2	0.756
AT5G14760	1271	0.757
AT5G18410	256	0.757
AT3G58610	1501	0.758
AT1G21690	833	0.758
AT1G07140	157	0.758
AT1G13560	1720	0.759
AT3G15290	70	0.759
AT4G12570	10	0.759
AT3G52660	1	0.759
AT4G33070	366	0.76
AT1G71500	59	0.76
AT1G77420	3	0.76
AT1G02840	2	0.76
AT1G78800	2	0.76
AT2G39940	15100	0.761
AT2G16850	6	0.761
AT3G22890	84	0.762

AT2G03420	3	0.762
AT5G44780	1	0.762
AT1G68850	94	0.763
AT4G23140	76	0.763
AT1G18070	8902	0.764
AT1G67500	19	0.764
AT1G52380	16	0.764
AT3G57040	14	0.765
AT5G56710	351	0.766
*AT4G39800	44	0.766
AT5G14650	7	0.766
AT4G28450	1	0.767
*AT2G44950	893	0.769
AT4G25660	229	0.769
AT3G15980	5	0.769
AT1G64790	1	0.769
AT4G33865	1530	0.77
*AT5G23120	46	0.77
AT4G26570	91	0.771
AT3G04740	676	0.772
AT1G32230	2	0.772
AT5G62530	1	0.773
AT4G33220	11	0.774
AT4G02450	2	0.775
AT5G20850	2215	0.776
*AT5G65800	1239	0.776
AT3G27820	1	0.776
AT1G01370	17	0.777
AT4G21710	2628	0.779
AT3G15352	434	0.779
AT3G54620	2	0.779
AT1G13870	1	0.779
AT1G22770	2784	0.78
AT3G21510	2068	0.78
AT1G77120	378	0.782
AT5G09260	836	0.784
*AT1G79280	26	0.784
AT5G08330	3	0.785
AT2G30280	26	0.786
AT3G55980	1	0.786
AT5G55990	10538	0.787
AT5G11770	36	0.788
AT5G05670	7	0.788

AT4G16520	11	0.79
*AT3G25100	2033	0.791
AT5G54260	1931	0.791
AT2G37340	6	0.791
AT2G45660	4	0.791
AT1G64750	2795	0.792
AT3G11410	41	0.792
AT1G49340	46	0.793
AT3G05230	14	0.793
AT1G53030	2	0.793
AT5G45900	37	0.794
AT2G47980	7	0.794
AT4G18230	1	0.794
AT5G22920	6	0.796
*AT2G30970	242	0.797
AT4G23630	12	0.797
AT2G23930	3	0.797
AT4G38160	2	0.797
AT1G07840	203	0.798
AT1G61340	1	0.798
AT5G12370	1209	0.8
AT5G52520	6	0.801
AT4G36480	9	0.802
AT1G02010	2	0.802
AT3G30775	174	0.803
AT2G34480	44	0.803
AT3G01340	12	0.803
AT4G24690	11	0.803
AT3G58490	195	0.804
AT1G09320	2	0.804
AT5G26360	6	0.805
AT3G05520	5	0.806
AT1G44900	627	0.807
AT1G48930	26	0.808
AT2G43950	15	0.808
AT1G30870	1	0.808
AT4G39400	6819	0.809
AT1G49180	400	0.809
AT1G17410	155	0.809
AT5G61600	8	0.809
AT3G09200	631	0.81
AT1G17050	3	0.81
AT4G26970	3	0.811

AT5G39400	1	0.812
AT1G32640	3110	0.813
AT1G74050	8	0.813
AT3G17510	9935	0.814
*AT5G50920	879	0.814
AT3G63460	75	0.815
AT5G03080	188	0.816
*AT2G36880	65	0.816
AT2G33560	5	0.816
*AT5G56290	3817	0.818
AT1G43170	3380	0.818
AT3G12250	17	0.818
AT1G18660	2	0.818
AT3G01990	1	0.819
AT1G10030	49	0.821
AT1G71790	2	0.821
AT4G22970	1	0.822
AT4G16110	36	0.823
AT4G20280	31	0.823
AT5G01220	1536	0.824
ATCG01110	282	0.824
AT2G28880	6	0.824
AT2G17520	5	0.824
AT5G57380	707	0.825
AT1G63810	124	0.825
AT2G01120	18	0.825
AT3G26900	1	0.825
AT5G01630	35	0.828
AT3G47470	10	0.828
*AT1G30000	1	0.828
AT4G26450	7	0.829
AT5G16560	11	0.83
AT3G26320	8	0.83
AT4G16340	250	0.831
AT5G16320	24	0.831
AT1G52930	3490	0.832
AT4G35530	328	0.832
AT1G48630	34	0.834
AT3G53580	6	0.834
AT1G19170	2	0.837
AT2G43210	2	0.837
AT3G54630	7	0.838
AT4G27330	3	0.838

AT2G30410	1	0.838
AT5G16130	200	0.841
AT3G54860	78	0.841
AT3G19720	41	0.841
AT5G03500	29	0.841
ATCG00180	811	0.842
AT3G01090	59	0.842
AT1G75820	5945	0.843
AT4G35950	68	0.843
AT4G29060	6	0.843
AT5G04920	831	0.845
AT3G51300	5	0.845
*AT4G10450	1139	0.846
AT3G48610	8	0.846
*AT1G73910	1	0.846
AT1G52220	211	0.847
*AT5G38420	197	0.847
AT1G26570	115	0.847
AT5G01640	36	0.847
AT5G54080	496	0.848
AT3G07850	22	0.848
AT1G27300	5341	0.849
AT1G60080	984	0.849
AT5G05590	771	0.849
AT2G42120	396	0.85
*AT2G46830	748	0.851
AT1G26665	11	0.851
AT5G17990	772	0.852
AT2G26230	131	0.852
AT2G28800	101	0.852
AT3G13222	11	0.852
AT3G25980	4445	0.853
AT5G16840	246	0.853
AT3G09630	30	0.853
AT2G21940	12	0.853
AT1G71820	1065	0.854
AT3G61190	28	0.854
AT5G08050	2	0.854
AT4G33030	1530	0.856
AT5G48010	45	0.856
AT4G16360	13	0.856
AT1G12060	1	0.856
AT3G28730	52	0.857

AT1G30970	24	0.857
AT3G21540	2	0.857
AT4G24820	11122	0.858
AT4G16845	707	0.858
AT3G13170	66	0.858
AT5G59950	38	0.858
AT2G34980	18	0.858
AT3G08740	12	0.858
AT1G02970	707	0.86
*AT5G42190	803	0.861
AT1G14850	469	0.861
AT1G23420	3	0.861
AT4G11280	27	0.862
AT1G29990	1	0.863
AT4G38630	5596	0.864
AT3G16310	442	0.864
AT1G63160	408	0.864
AT1G12360	1062	0.865
AT2G37090	1	0.865
AT4G14540	127	0.866
AT3G47620	680	0.867
AT3G16250	50	0.867
AT3G19590	4392	0.868
AT1G66670	54	0.868
AT3G54180	16	0.868
AT2G47610	151	0.87
AT5G26710	4484	0.871
AT3G58170	213	0.871
AT3G10970	16	0.871
AT3G19290	4	0.872
AT2G21440	3	0.872
*AT3G54610	383	0.873
AT4G32910	114	0.874
AT1G44170	2	0.874
AT1G70290	6	0.875
AT2G44690	290	0.876
AT2G44050	2	0.876
*AT1G54360	2237	0.877
AT1G12480	975	0.877
AT1G09020	188	0.877
AT1G16240	857	0.878
AT1G55190	14	0.878
AT1G68760	2	0.878

AT1G27840	2095	0.879
AT3G53190	52	0.88
AT3G06650	46	0.88
AT1G59980	8	0.88
AT2G20690	1	0.88
*AT4G13930	1179	0.881
*AT1G03130	268	0.881
*AT1G33140	120	0.881
AT3G62420	8	0.881
AT5G28740	2233	0.882
AT3G25520	1	0.882
AT5G67100	1599	0.884
AT3G12480	121	0.884
AT4G32190	57	0.884
AT3G18600	26	0.886
AT4G02610	1351	0.887
AT3G59220	13	0.887
AT5G28770	2	0.887
AT2G20635	1	0.888
AT1G30825	8	0.889
AT4G01370	5999	0.89
AT4G35580	447	0.89
AT5G64270	291	0.89
AT3G07370	64	0.89
AT1G11750	55	0.891
AT5G45390	54	0.891
AT4G18040	2348	0.892
AT4G12720	3	0.892
AT1G77350	3	0.893
AT4G02020	707	0.894
*AT3G27000	44	0.894
AT5G45775	7	0.894
AT1G01140	26	0.895
AT5G11520	2258	0.896
AT3G59050	2	0.896
AT1G11920	38	0.897
AT3G57260	30	0.898
AT2G39800	1269	0.899
AT5G46860	78	0.899
AT3G15720	36	0.899
AT1G75040	23	0.899
ATCG00170	801	0.9
AT1G15980	14	0.9

AT4G02510	19	0.901
AT1G49540	1	0.901
AT4G40042	840	0.902
*AT4G34390	1	0.902
AT3G14390	1	0.902
AT5G14060	23	0.903
AT3G55340	5	0.903
AT3G14120	703	0.905
AT3G56640	85	0.905
AT3G54840	13	0.905
AT1G24807	7	0.905
AT3G12400	827	0.906
AT3G46060	13	0.906
*AT5G26310	9	0.906
AT5G58180	2339	0.907
AT4G01560	14	0.907
AT5G20320	3	0.907
*AT2G05990	1	0.907
*AT2G27710	1870	0.908
AT3G27530	35	0.908
AT4G29810	5733	0.909
AT4G02640	1	0.909
*AT5G18170	1175	0.91
AT5G66540	21	0.91
AT5G60120	5	0.91
AT2G03410	1	0.91
AT5G26980	2348	0.911
AT5G53350	12	0.911
AT2G20050	369	0.912
AT3G45100	6	0.913
AT3G42660	1	0.914
AT5G48220	772	0.916
*AT2G38080	17	0.916
AT1G01560	6	0.916
AT1G80670	1161	0.917
AT5G04240	15	0.917
AT1G23740	6	0.917
AT5G27420	2	0.917
AT5G19280	5931	0.918
AT5G20020	5	0.918
AT4G20380	1	0.918
AT2G32220	767	0.919
AT5G08080	358	0.919

AT1G07660	209	0.92
AT3G56990	23	0.92
AT4G25200	2	0.92
AT5G41600	82	0.921
AT2G29530	8	0.921
AT1G25260	5891	0.922
AT2G30050	413	0.922
AT1G79990	7	0.922
AT3G10530	465	0.923
AT3G54280	121	0.923
AT2G14610	7	0.923
AT5G27850	8561	0.924
AT3G19100	839	0.925
AT2G18950	25	0.925
AT5G63110	2	0.926
AT3G13970	4	0.927
AT5G20010	84	0.928
AT3G08950	7	0.928
AT3G07010	3	0.928
AT5G20070	6	0.929
*AT2G27040	27	0.93
AT1G23820	2	0.93
AT1G23860	6	0.931
AT5G63610	676	0.934
AT3G04400	226	0.934
AT1G26670	32	0.934
*AT2G26080	21414	0.937
AT5G19990	974	0.937
AT3G45640	332	0.938
AT1G06720	9	0.939
AT2G28900	2	0.939
AT5G13680	1	0.939
*AT3G17240	25624	0.94
AT1G22790	6	0.94
AT5G05680	5	0.94
AT2G04630	134	0.941
ATCG00140	14	0.941
AT1G22750	6	0.941
AT5G48650	4	0.941
AT3G01270	1	0.941
AT1G75350	516	0.942
AT3G51800	129	0.942
AT1G74340	22	0.942

AT5G23880	2368	0.943
AT3G52430	11	0.943
AT1G63660	12576	0.944
AT5G13960	22	0.944
AT1G61580	3	0.945
AT3G06850	4419	0.946
AT2G31970	1522	0.946
AT4G25740	1311	0.947
AT3G29350	72	0.947
AT5G52100	10	0.947
AT1G15130	2	0.947
AT5G14250	74873	0.948
AT4G12600	4705	0.948
AT2G45640	364	0.949
AT2G19520	104	0.949
AT1G80840	5	0.951
AT5G56860	3	0.951
AT2G46225	10	0.953
AT1G02410	7	0.953
AT2G44860	654	0.954
AT4G29170	70	0.955
AT1G48760	6	0.956
AT5G37510	26271	0.958
AT1G71830	7407	0.958
AT4G15900	41	0.958
AT5G58760	10612	0.959
AT3G48150	9104	0.959
AT2G36900	269	0.959
AT1G60140	223	0.959
*AT5G02500	8	0.959
AT3G11400	56748	0.96
AT3G57530	8	0.96
AT1G11400	1	0.96
AT2G16440	1833	0.961
AT4G29840	962	0.961
AT5G45130	48	0.961
AT5G16630	10960	0.962
AT5G44200	2748	0.962
AT2G17265	946	0.962
AT5G60460	93	0.962
AT1G34130	3	0.962
AT3G11870	2	0.962
AT3G17860	1642	0.963

AT3G50670	1570	0.963
AT4G13780	1136	0.963
AT4G00100	3	0.963
AT1G15440	2397	0.964
AT1G13980	18	0.964
AT3G53430	3	0.964
AT3G06530	9684	0.965
AT4G24040	239	0.965
AT2G42270	1	0.965
*AT4G31120	181	0.966
AT1G77470	106	0.966
AT1G04170	42	0.966
AT3G53610	18	0.966
AT1G49880	8	0.966
AT2G39630	5	0.966
AT5G23395	546	0.967
*AT3G63490	5	0.967
AT4G31480	3	0.967
AT1G18270	2617	0.968
*AT3G55280	2348	0.968
AT1G06570	1424	0.968
AT1G02080	3	0.969
AT4G14365	1	0.969
AT5G64813	1	0.969
AT4G31720	394	0.97
AT4G36690	9	0.97
AT4G30820	7	0.971
AT1G17530	915	0.972
AT5G47010	5123	0.973
AT1G63780	16	0.973
AT5G52640	697	0.974
AT5G22290	611	0.974
AT4G16280	107	0.974
AT1G10390	22	0.975
AT1G55610	4	0.975
AT2G17930	157	0.976
AT4G17270	2	0.976
AT5G50810	246	0.977
AT5G58860	38	0.977
AT5G43330	6	0.977
AT2G42260	1	0.977
AT3G46560	524	0.978
AT1G25490	227	0.978

AT1G31540	70	0.978
AT5G17020	52	0.978
AT3G61240	1	0.978
AT4G17190	1	0.978
AT5G50320	1	0.978
AT1G16350	11795	0.979
AT1G51510	318	0.979
AT2G35110	10	0.979
AT4G19003	5	0.979
AT1G01630	2	0.979
AT1G28490	1278	0.98
AT4G34450	963	0.98
AT4G11110	940	0.98
AT2G36160	93	0.98
ATCG00130	88	0.98
AT1G60550	7	0.98
AT5G59240	6	0.98
AT3G04840	5157	0.981
AT5G16850	2917	0.981
AT3G52730	677	0.981
AT1G49850	3	0.981
*AT4G26070	2	0.981
AT2G38910	2	0.981
AT1G54140	1452	0.982
AT1G20960	553	0.982
AT1G80070	431	0.982
AT1G76400	105	0.982
AT5G11170	2	0.982
AT1G02090	71400	0.983
AT3G18524	269	0.983
AT5G42590	17	0.983
AT5G05760	3298	0.984
AT5G57870	31	0.984
AT5G16830	316	0.985
AT1G77840	29	0.985
*AT1G30270	3311	0.986
AT2G26650	3168	0.986
AT5G61210	2492	0.986
AT4G02060	1789	0.986
ATCG00120	115	0.986
AT1G05070	44	0.986
AT1G66070	37	0.986
AT3G05590	10	0.986

AT2G07690	3	0.986
AT5G15680	1	0.986
AT1G08560	2012	0.987
AT5G17170	13	0.987
AT4G27040	5	0.987
AT5G66690	4	0.987
AT2G43370	1	0.987
AT3G63410	1	0.987
AT1G56110	10768	0.988
AT3G02090	9061	0.988
AT2G04660	5119	0.988
AT1G03950	5	0.988
AT2G31500	4	0.988
AT1G17760	1143	0.989
AT1G16610	591	0.989
AT3G08530	4	0.99
AT3G50920	8	0.991
AT3G12990	1	0.991
AT1G72370	11563	0.992
AT1G61570	923	0.992
AT5G14050	68	0.992
AT3G47070	23	0.992
*AT5G13450	21	0.992
AT3G16320	4024	0.993
AT5G14520	3393	0.993
AT3G10050	979	0.993
AT5G13480	490	0.993
AT4G34460	62	0.993
AT1G13730	6	0.993
AT1G72560	2	0.993
AT4G37490	4024	0.994
AT3G11820	2255	0.994
AT4G32650	2245	0.994
AT2G40360	1197	0.994
AT1G69070	1166	0.994
AT2G45070	1040	0.994
AT2G40290	8	0.994
AT3G48190	8	0.994
AT2G33120	4	0.994
AT5G05470	2377	0.995
AT1G44110	446	0.995
AT4G34700	74	0.995
AT4G28200	68	0.995

AT3G11590	3	0.995
AT3G24500	2	0.995
AT4G17770	2	0.995
AT1G17440	1	0.995
AT5G20290	13423	0.996
AT1G32750	4530	0.996
AT5G63960	2278	0.996
AT1G20580	2120	0.996
AT1G17980	36	0.996
AT4G31500	9	0.996
AT1G10580	8	0.996
AT4G39950	8	0.996
AT5G41880	6	0.996
AT1G63990	2	0.996
AT1G23360	1	0.996
AT2G43790	5764	0.997
AT3G12580	714	0.997
AT3G60360	399	0.997
AT5G53970	132	0.997
AT2G35190	107	0.997
AT3G60770	48	0.997
AT1G27720	14	0.997
AT5G66130	8	0.997
AT3G43520	7	0.997
AT2G35890	4	0.997
AT2G45000	3	0.997
AT4G17380	2	0.997
AT5G59180	1572	0.998
AT5G51660	1414	0.998
AT1G09760	243	0.998
AT1G68890	25	0.998
AT5G15750	13	0.998
AT3G54640	10	0.998
AT1G25220	5	0.998
AT5G66210	4	0.998
AT1G14340	1	0.998
AT3G13230	1	0.998
AT3G57290	73308	0.999
AT3G46940	3603	0.999
*AT3G09840	3211	0.999
AT3G27240	1789	0.999
AT1G67630	1670	0.999
AT2G15430	1390	0.999

*AT2G38470	1132	0.999
AT1G11475	1124	0.999
AT2G44520	154	0.999
AT3G17910	149	0.999
ATMG01360	129	0.999
AT1G74710	24	0.999
AT2G07687	21	0.999
AT1G60600	18	0.999
AT2G29210	11	0.999
AT5G05010	10	0.999
AT4G11420	3	0.999
AT4G09140	2	0.999
AT4G38130	1	0.999
AT3G48750	44681	1
AT4G25630	22092	1
AT2G29680	21555	1
AT5G27620	15405	1
AT5G58290	15270	1
AT3G59540	12477	1
AT1G66750	11754	1
AT4G35800	11445	1
AT3G56150	7728	1
AT3G05060	7299	1
AT1G61700	6886	1
AT1G60850	6716	1
AT3G57660	6700	1
AT2G32060	6442	1
AT3G22320	6005	1
AT1G07370	5762	1
AT3G60240	5656	1
AT4G12610	5525	1
AT5G46280	5035	1
AT2G13540	4588	1
AT1G18340	3985	1
AT4G30220	3655	1
AT3G57150	3071	1
AT1G03190	2629	1
AT3G11964	2186	1
AT1G33980	2122	1
AT5G50460	1678	1
AT1G29940	1410	1
AT5G08180	1331	1
AT5G06160	1311	1

AT2G32600	1187	1
AT5G25780	1103	1
AT1G31660	865	1
AT5G41150	738	1
AT3G28030	704	1
AT4G17750	448	1
AT1G55520	414	1
AT5G09920	239	1
AT3G04920	228	1
AT1G49950	183	1
AT2G05210	183	1
AT3G03920	50	1
AT3G20780	32	1
AT5G02820	31	1
AT3G23620	27	1
AT1G22840	22	1
AT3G48090	21	1
AT1G24280	19	1
AT5G43470	15	1
*AT1G48850	5	1
AT2G25830	5	1
AT2G07695	4	1
AT5G25450	4	1
AT4G37360	2	1

*: validated gene
