# Supplementary material for: A Shortest-Path-Based Method for the Analysis and Prediction of Fruit-Related Genes in Arabidopsis thaliana
Source: PLoS One. 2016 Jul 19;11(7):e0159519. doi: 10.1371/journal.pone.0159519 (PMC4951011; doi:10.1371/journal.pone.0159519)
Supplement: S6 Table — (PDF) [file pone.0159519.s006.pdf]

S6 Table. 653 genes filtering by a permutation test, where 136 are validated genes and 517 are novel genes.

(1) 136 validated genes

Ensembl ID	Betweenness	Permutation FDR
AT5G63310	83568	<0.001
AT1G75950	62597	<0.001
AT5G57360	57180	<0.001
AT1G23190	37375	<0.001
AT5G20620	36293	<0.001
AT1G11860	33530	<0.001
AT2G39730	33121	<0.001
AT3G03250	32867	<0.001
AT5G42740	32068	<0.001
AT3G55800	30025	<0.001
AT4G38970	23445	<0.001
AT5G48300	22711	<0.001
AT5G09660	21995	<0.001
AT1G79550	16680	<0.001
AT1G14700	16066	<0.001
AT1G42970	13104	<0.001
AT2G45790	10993	<0.001
AT3G63010	10227	<0.001
AT2G36530	9898	<0.001

AT1G15550	9317	<0.001
AT4G29130	9150	<0.001
AT3G54660	6577	<0.001
AT5G20720	6556	<0.001
AT1G04410	5308	<0.001
AT3G17790	3765	<0.001
AT2G01890	1990	<0.001
AT4G10340	1901	<0.001
AT5G24300	1802	<0.001
AT1G13440	1682	<0.001
AT1G04420	1530	<0.001
AT3G01500	548	<0.001
AT3G53460	311	<0.001
AT2G21660	297	<0.001
AT5G58330	32	<0.001
AT5G03630	21	<0.001
AT1G02205	17	<0.001
AT1G76430	13	<0.001
AT1G52400	9	<0.001
AT1G10010	2	<0.001
AT2G40100	1	<0.001
AT4G09320	6259	0.001

AT3G09820	3032	0.001
AT2G45240	2738	0.001
AT3G62710	1716	0.001
AT5G17920	640	0.001
AT5G07200	17	0.001
AT5G64570	6	0.001
AT1G75110	1	0.001
AT3G06580	4276	0.002
AT3G54890	1523	0.002
AT3G59990	1	0.002
AT1G06680	2142	0.003
AT4G01060	1135	0.003
AT5G49360	1069	0.003
AT5G01410	2081	0.004
AT3G50820	1876	0.004
AT4G13940	1622	0.004
AT3G19700	985	0.004
AT4G38320	2	0.004
AT2G33150	23842	0.005
AT1G07890	3338	0.005
AT2G42620	1960	0.005
AT1G72990	918	0.005

AT2G44180	1025	0.006
AT2G32770	6	0.006
AT3G60750	3654	0.007
AT3G20500	1799	0.007
AT3G04120	1024	0.007
AT1G01610	228	0.007
AT5G48580	15	0.007
AT4G33010	36856	0.008
AT1G32060	2260	0.008
AT5G19220	394	0.008
AT1G14870	289	0.009
AT1G76450	145	0.009
AT1G53510	1	0.009
AT4G18480	2249	0.01
AT2G35980	7	0.01
AT1G75090	1	0.01
AT1G68560	1265	0.011
AT5G23720	985	0.012
AT5G20830	188	0.012
AT1G68530	3070	0.013
AT4G28750	2239	0.013
AT3G15020	314	0.013

AT1G32470	9521	0.014
AT3G26060	2891	0.014
AT3G13470	986	0.014
AT5G63840	3685	0.015
AT5G42310	32	0.016
AT5G63140	7	0.016
AT4G28190	1	0.016
AT2G40220	2871	0.017
AT1G23730	2516	0.017
AT2G36305	846	0.017
AT3G52820	1	0.017
AT5G38410	986	0.021
AT3G46440	1	0.021
AT5G49910	1	0.021
AT4G25470	955	0.022
AT2G46070	925	0.022
AT1G73360	1	0.023
AT3G51160	1674	0.024
AT1G05160	23	0.025
AT3G55510	945	0.027
AT2G06050	1747	0.028
AT2G28000	955	0.028

AT3G10920	913	0.028
AT3G57610	7842	0.03
AT1G09340	1520	0.03
AT1G75750	4	0.03
AT4G03520	1	0.03
AT3G55330	4686	0.031
AT4G23670	372	0.032
AT3G23990	1913	0.033
AT5G42020	3699	0.034
AT1G31740	1	0.034
AT4G39260	538	0.035
AT5G11450	101	0.037
AT2G24200	4	0.041
AT1G22640	1	0.041
AT5G60540	987	0.042
AT5G57140	900	0.042
AT1G50900	892	0.042
AT5G53560	465	0.042
AT1G20330	167	0.042
AT4G09650	1852	0.043
AT2G40490	2061	0.044
AT2G18915	1445	0.044

AT1G53500	870	0.044
AT2G21330	500	0.044
AT5G14740	129	0.044
AT3G12780	5514	0.045
AT3G45140	91	0.048
AT5G48810	2	0.048
AT3G21160	4	0.049

(2) 517 novel genes

Ensembl ID	Betweenness	Permutation FDR
AT1G09570	81073	<0.001
AT5G59440	64367	<0.001
AT2G18790	62183	<0.001
AT4G02570	61598	<0.001
AT4G29040	33682	<0.001
AT5G09900	30006	<0.001
AT2G42790	26876	<0.001
AT3G54050	26451	<0.001
AT5G18200	20382	<0.001
AT2G01570	17034	<0.001
AT5G51820	13210	<0.001
AT1G67070	11838	<0.001
AT2G39770	10490	<0.001

AT2G43010	9400	<0.001
AT4G16760	8472	<0.001
AT1G55490	8405	<0.001
AT3G02870	8284	<0.001
AT1G49430	7455	<0.001
AT3G22960	6812	<0.001
AT1G44575	6635	<0.001
AT4G26850	6277	<0.001
ATCG00490	5787	<0.001
AT2G38280	4938	<0.001
AT1G79470	4472	<0.001
AT1G10970	3985	<0.001
AT2G27150	3954	<0.001
AT1G47220	3754	<0.001
AT1G52410	3717	<0.001
AT1G75080	3580	<0.001
AT5G27380	3222	<0.001
AT1G32340	2958	<0.001
AT2G44160	2322	<0.001
AT1G24470	2083	<0.001
AT4G25420	1952	<0.001
AT5G60440	1946	<0.001

AT2G35690	1840	<0.001
AT3G26090	1838	<0.001
AT1G20510	1812	<0.001
AT2G02230	1585	<0.001
AT1G13590	1552	<0.001
AT4G08870	1374	<0.001
AT2G36310	998	<0.001
AT5G01650	988	<0.001
AT2G37020	987	<0.001
AT3G29810	961	<0.001
AT4G20930	911	<0.001
AT5G61130	891	<0.001
AT3G27810	773	<0.001
AT2G07725	729	<0.001
AT5G57410	588	<0.001
AT1G50940	480	<0.001
AT1G13580	445	<0.001
AT3G02040	180	<0.001
AT3G26220	122	<0.001
AT1G77490	118	<0.001
AT5G52920	114	<0.001
AT2G34770	108	<0.001

AT1G75120	87	<0.001
AT5G44640	81	<0.001
AT5G06720	80	<0.001
AT3G13700	67	<0.001
AT4G30920	60	<0.001
AT1G80340	24	<0.001
AT5G06280	14	<0.001
AT5G43300	14	<0.001
AT2G18130	13	<0.001
AT3G48670	12	<0.001
AT4G21000	8	<0.001
AT4G24460	8	<0.001
AT3G15990	6	<0.001
AT2G28210	5	<0.001
AT1G17820	2	<0.001
AT5G64750	2	<0.001
AT1G74458	1	<0.001
AT4G29600	1	<0.001
AT4G37720	1	<0.001
AT5G32475	1	<0.001
AT5G51550	1	<0.001
AT5G61420	1	<0.001

AT1G09530	19548	0.001
AT1G32100	4742	0.001
AT1G20630	4129	0.001
AT5G61790	3866	0.001
AT3G49250	3842	0.001
AT1G22300	3292	0.001
AT2G35635	1891	0.001
AT3G23000	1842	0.001
AT3G25780	1804	0.001
AT2G22860	1491	0.001
AT5G23900	830	0.001
AT1G06180	493	0.001
AT4G24160	476	0.001
AT1G24620	18	0.001
AT5G10560	13	0.001
AT4G23750	4	0.001
AT1G10460	1	0.001
AT2G32690	1	0.001
AT2G33510	1	0.001
AT3G28430	1	0.001
AT4G10260	1	0.001
AT4G32480	1	0.001

AT5G58080	1	0.001
AT4G24620	12056	0.002
AT3G20770	6439	0.002
AT4G35090	5729	0.002
AT5G25900	3651	0.002
AT3G23490	3226	0.002
AT4G25100	3027	0.002
AT3G47930	2989	0.002
AT4G17360	1973	0.002
AT2G37260	1608	0.002
AT4G23100	1095	0.002
AT4G38900	973	0.002
AT1G55920	210	0.002
AT5G54650	49	0.002
AT3G42830	10	0.002
AT1G17860	7	0.002
AT2G01735	3	0.002
AT5G57540	1	0.002
AT2G36270	9184	0.003
AT5G03730	1975	0.003
AT1G49005	978	0.003
AT3G51590	966	0.003

AT3G59060	640	0.003
AT2G15340	158	0.003
AT3G25760	51	0.003
AT2G47240	35	0.003
AT5G02630	5	0.003
AT3G08770	4	0.003
AT3G16400	3	0.003
AT5G54310	3	0.003
AT3G18180	2	0.003
AT3G28970	2	0.003
AT3G46120	1	0.003
AT3G53550	1	0.003
AT4G18910	1	0.003
AT5G39160	1	0.003
AT3G24650	10673	0.004
AT2G25490	6353	0.004
AT1G67490	4607	0.004
AT1G30620	1910	0.004
AT1G64440	1759	0.004
AT2G18850	990	0.004
AT2G20180	988	0.004
AT4G12920	987	0.004

AT1G71270	296	0.004
AT1G02390	4	0.004
AT1G69870	2	0.004
AT2G02850	2	0.004
AT1G65730	1	0.004
AT3G03660	1	0.004
AT5G42400	1	0.004
AT3G61140	4897	0.005
AT5G08280	3407	0.005
AT5G26030	1808	0.005
AT4G37040	1081	0.005
AT1G14400	990	0.005
AT1G12900	496	0.005
AT1G21560	24	0.005
AT4G04950	16	0.005
AT5G59030	6	0.005
AT2G19500	4	0.005
AT1G29980	3	0.005
AT4G18870	2	0.005
AT1G65090	1	0.005
AT1G80620	1	0.005
AT2G14910	1	0.005

AT2G25150	1	0.005
AT5G15310	1	0.005
AT5G39130	1	0.005
AT1G52340	4070	0.006
AT3G26744	3715	0.006
AT2G28550	742	0.006
AT1G74030	98	0.006
AT3G12460	21	0.006
AT4G09760	9	0.006
AT3G60390	8	0.006
AT1G63260	3	0.006
AT4G25900	3	0.006
AT5G57740	1	0.006
AT5G59700	1	0.006
AT2G37040	9204	0.007
AT2G18740	2395	0.007
AT4G25120	1412	0.007
AT4G29640	966	0.007
AT5G44480	848	0.007
AT2G31800	37	0.007
AT1G78000	14	0.007
AT1G11260	6	0.007

AT2G34130	2	0.007
AT5G25480	2	0.007
AT1G50280	1	0.007
AT4G27430	3670	0.008
AT4G14910	2943	0.008
AT2G34555	988	0.008
AT3G57230	960	0.008
AT3G59380	459	0.008
AT5G41000	28	0.008
AT1G15350	1	0.008
AT5G61380	18437	0.009
AT3G13120	9887	0.009
AT4G01690	2804	0.009
AT1G71340	1391	0.009
AT5G58730	1023	0.009
AT4G03510	982	0.009
AT5G22875	533	0.009
AT1G78050	42	0.009
AT5G04800	5	0.009
AT5G43980	5	0.009
AT1G64405	4	0.009
AT1G03475	2681	0.01

AT5G56580	1904	0.01
AT1G20260	1503	0.01
AT1G04300	987	0.01
AT5G67110	980	0.01
AT1G19200	3	0.01
AT5G13350	2	0.01
AT5G25250	2	0.01
AT1G49190	1	0.01
AT4G09610	1	0.01
AT1G75330	10728	0.011
AT1G69120	8610	0.011
AT5G10480	7316	0.011
AT1G24260	7001	0.011
AT3G29360	4377	0.011
AT2G02500	1763	0.011
AT4G02195	1064	0.011
AT1G79040	365	0.011
AT1G04880	1	0.011
AT5G20570	46365	0.012
AT3G54470	10270	0.012
AT5G35530	7739	0.012
AT3G03780	2030	0.012

AT1G78630	1809	0.012
AT3G46580	981	0.012
AT1G56430	6	0.012
AT4G12750	1	0.012
AT4G31550	1	0.012
AT2G34590	8952	0.013
AT5G47120	1057	0.013
AT3G03700	985	0.013
AT3G55150	711	0.013
AT1G69820	151	0.013
AT3G54830	3	0.013
AT3G03080	1	0.013
AT4G36260	1	0.013
AT1G01040	2062	0.014
AT3G54710	1530	0.014
AT3G21070	985	0.014
AT3G22600	131	0.014
AT5G02780	29	0.014
AT5G57720	4	0.014
AT1G17120	1	0.014
AT1G70330	1	0.014
AT5G52390	1	0.014

AT1G76030	9854	0.015
AT1G25350	1235	0.015
AT1G34790	992	0.015
AT1G24510	5	0.015
AT3G01140	1	0.015
AT5G22860	1	0.015
AT1G28300	1264	0.016
AT5G09640	993	0.016
AT5G10420	2	0.016
AT4G24830	11908	0.017
AT3G19820	3607	0.017
AT1G48410	2059	0.017
AT5G04360	981	0.017
AT1G09870	8	0.017
AT4G26710	6	0.017
AT3G12470	1	0.017
AT3G29320	3567	0.018
AT1G69740	3370	0.018
AT1G26945	988	0.018
AT5G17050	988	0.018
AT3G45070	947	0.018
AT4G12430	11	0.018

AT5G09978	4	0.018
AT5G24900	3	0.018
AT2G28100	2	0.018
AT1G01900	1	0.018
AT5G64860	1964	0.019
AT1G52980	971	0.019
AT2G28190	916	0.019
AT1G15210	5	0.019
AT3G29770	2	0.019
AT4G13770	1	0.019
AT1G21970	2229	0.02
AT5G47700	936	0.02
AT1G08630	155	0.02
AT5G42000	25	0.02
AT1G71300	14	0.02
AT5G51760	9	0.02
AT5G58270	3	0.02
AT5G19690	5576	0.021
AT4G26300	1086	0.021
AT1G66280	182	0.021
AT2G25900	9	0.021
AT1G54280	4	0.021

AT3G24503	696	0.022
AT5G09420	20	0.022
AT3G55360	5320	0.023
AT3G56940	1990	0.023
AT2G19940	1956	0.023
AT2G38620	1922	0.023
AT2G10940	1243	0.023
AT4G21270	1027	0.023
AT3G48000	988	0.023
AT3G05010	987	0.023
AT1G68650	28	0.023
AT3G49260	2	0.023
AT1G01960	1	0.024
AT5G50010	1	0.024
AT1G78300	3719	0.025
AT2G26930	1758	0.025
AT3G54440	1511	0.025
AT1G78955	9	0.025
AT2G37060	2	0.025
AT1G74810	1	0.025
AT3G21110	1009	0.026
AT1G04550	971	0.026

AT1G05820	17	0.026
AT2G26680	2	0.026
AT4G08950	2	0.026
AT1G05470	322	0.027
AT1G65360	14	0.027
AT1G67940	6	0.027
AT4G22930	7284	0.028
AT2G40030	4770	0.028
AT5G48230	1838	0.028
AT1G01280	968	0.028
AT5G55250	12	0.028
AT5G28646	4	0.028
AT1G20380	2	0.028
AT4G18395	2	0.028
AT4G33670	2938	0.029
AT4G14700	1891	0.029
AT4G02530	1192	0.029
AT1G14420	620	0.029
AT2G15640	559	0.029
AT3G54210	214	0.029
AT5G17770	158	0.029
AT1G22710	2	0.029

AT3G62980	16158	0.03
AT3G48730	3526	0.03
AT5G17220	2126	0.03
AT2G40380	991	0.03
AT1G42550	985	0.03
AT4G32640	186	0.03
AT1G21230	163	0.03
AT5G02010	8	0.03
AT5G25390	1	0.03
AT4G28660	1967	0.031
AT1G73720	1010	0.031
AT1G56050	981	0.031
AT1G13370	555	0.031
AT2G20670	15	0.031
AT3G07390	5	0.031
AT4G26320	3	0.031
AT2G02770	1	0.031
AT5G60410	7775	0.032
AT4G03415	976	0.032
AT5G40280	454	0.032
AT5G41080	236	0.032
AT5G20250	9	0.032

AT4G26910	6349	0.033
AT1G74210	1375	0.033
AT5G47930	1220	0.033
AT4G32551	1030	0.033
AT4G17870	983	0.033
AT5G20840	968	0.033
AT3G12410	963	0.033
AT4G08770	278	0.033
AT3G63200	35	0.033
AT5G39110	3	0.033
AT2G39450	2	0.033
AT5G19550	28118	0.034
AT5G47030	1517	0.034
AT5G10240	971	0.034
AT2G27030	949	0.034
AT2G30810	260	0.034
AT2G43360	6	0.034
AT4G16690	2	0.034
AT1G09790	1	0.034
AT4G19420	1	0.034
AT1G03630	2072	0.035
AT5G66005	1627	0.035

AT4G32140	946	0.035
AT1G09310	855	0.035
AT4G16130	516	0.035
AT4G00540	46	0.035
AT3G19050	14	0.035
AT2G26170	3	0.035
AT3G13560	3	0.035
AT3G27580	2	0.035
AT3G04630	1	0.035
AT5G08690	5877	0.036
AT4G08900	4867	0.036
AT4G26900	3504	0.036
AT1G65060	1651	0.036
AT1G32990	1028	0.036
AT5G16760	980	0.036
AT5G66230	942	0.036
AT1G64290	244	0.036
AT2G03500	80	0.036
AT3G60620	34	0.036
AT2G01320	16	0.036
AT1G27950	8	0.036
AT2G16005	4	0.036

AT5G03280	2893	0.037
AT3G57800	973	0.037
AT3G55040	955	0.037
AT1G53750	939	0.037
AT2G42830	185	0.037
AT5G19940	132	0.037
AT1G47128	18	0.037
AT5G51750	3	0.037
AT5G03555	988	0.038
AT5G15170	924	0.038
AT4G19490	761	0.038
AT1G25230	633	0.038
AT1G16460	463	0.038
AT2G02860	241	0.038
AT1G21550	24	0.038
AT3G05810	1	0.038
AT3G47450	4249	0.039
AT1G55090	1003	0.039
AT3G01770	170	0.039
AT5G15190	47	0.039
AT5G56360	12	0.039
AT1G15820	6	0.039

AT1G09960	4	0.039
AT1G76590	1	0.039
AT3G48430	2393	0.04
AT4G09000	1777	0.04
AT5G66870	988	0.04
AT3G04720	475	0.04
AT3G14020	124	0.04
AT1G31190	118	0.04
AT2G36580	18	0.04
AT3G24310	17	0.04
AT5G35935	17	0.04
AT1G49670	1	0.04
AT1G65620	2700	0.041
AT3G13440	977	0.041
AT1G80260	976	0.041
AT4G14210	975	0.041
AT2G30000	444	0.041
AT1G24190	364	0.041
AT5G66220	294	0.041
AT4G29340	139	0.041
AT3G44540	6	0.041
AT1G19990	2	0.041

AT1G48900	860	0.042
AT3G16370	20	0.042
AT1G11960	18	0.042
AT2G38750	13	0.042
AT2G46650	11	0.042
AT5G05610	1	0.042
AT5G04040	404	0.043
AT4G35020	245	0.043
AT4G39700	186	0.043
AT4G16260	155	0.043
AT4G27440	19	0.043
AT5G64940	1	0.043
AT5G42800	8922	0.044
AT2G45170	907	0.044
AT2G27990	774	0.044
AT4G00720	346	0.044
AT1G02920	204	0.044
AT4G08390	8	0.044
AT2G39750	6	0.044
AT5G51570	4	0.044
AT3G54420	3	0.044
AT1G60110	1	0.044

AT4G35460	106	0.045
AT4G04070	82	0.045
AT4G34260	15	0.045
AT5G08530	6687	0.046
AT4G18960	1969	0.046
AT1G23310	1545	0.046
AT2G37620	957	0.046
AT5G55950	734	0.046
AT1G17070	694	0.046
AT5G20510	344	0.046
AT1G56145	17	0.046
AT5G05690	17	0.046
AT4G24510	9	0.046
AT2G34420	2	0.046
AT3G46110	1	0.046
AT3G59270	1	0.046
AT1G23290	797	0.047
AT3G61260	701	0.047
AT4G17730	622	0.047
AT5G40610	79	0.047
AT1G15910	12	0.047
AT3G25900	10	0.047

AT4G03090	2	0.047
AT2G33040	4057	0.048
AT4G36020	228	0.048
AT1G03970	16	0.048
AT4G16370	10	0.048
AT1G22500	1	0.048
AT3G54700	1	0.048
AT3G51240	2662	0.049
AT3G20000	2334	0.049
AT2G07050	1931	0.049
AT1G63290	1120	0.049
AT5G14220	997	0.049
AT3G21720	966	0.049
AT2G18770	595	0.049
AT5G17520	3	0.049
AT1G48020	2	0.049
AT1G14687	1	0.049
AT1G24520	1	0.049
AT4G28880	1	0.049
AT5G63530	1	0.049
